# A Single‐Nucleotide Mutation in a GLUTAMATE RECEPTOR‐LIKE Gene Confers Resistance to Fusarium Wilt in *Gossypium hirsutum*

**DOI:** 10.1002/advs.202002723

**Published:** 2021-02-19

**Authors:** Shiming Liu, Xiaojun Zhang, Shenghua Xiao, Jun Ma, Weijun Shi, Tao Qin, Hui Xi, Xinhui Nie, Chunyuan You, Zheng Xu, Tianyi Wang, Yujing Wang, Zhennan Zhang, Jianying Li, Jie Kong, Alifu Aierxi, Yu Yu, Keith Lindsey, Steven J. Klosterman, Xianlong Zhang, Longfu Zhu

**Affiliations:** ^1^ National Key Laboratory of Crop Genetic Improvement Huazhong Agricultural University Wuhan Hubei 430070 China; ^2^ Economic Crop Research Institute Xinjiang Academy of Agricultural Science Ürümqi Xinjiang 830091 China; ^3^ Key Laboratory of Oasis Ecology Agricultural of Xinjiang Bingtuan Agricultural College Shihezi University Shihezi Xinjiang 832000 China; ^4^ Cotton Research Institute Shihezi Academy of Agriculture Science Shihezi Xinjiang 832000 China; ^5^ Cotton Research Institute Xinjiang Academy of Agriculture and Reclamation Science Shihezi Xinjiang 832000 China; ^6^ Department of Biosciences Durham University Durham DH1 3LE UK; ^7^ Crop Improvement and Protection Research Unit USDA‐ARS Salinas CA 93905 USA

**Keywords:** disease‐resistant genes, Fusarium wilt, GLUTAMATE RECEPTOR‐LIKE genes, *Gossypium hirsutum*

## Abstract

Fusarium wilt (FW) disease of cotton, caused by the fungus *Fusarium oxysporum* f. sp. *vasinfectum* (*Fov*), causes severe losses in cotton production worldwide. Though significant advancements have been made in development of FW‐resistant Upland cotton (*Gossypium hirsutum*) in resistance screening programs, the precise resistance genes and the corresponding molecular mechanisms for resistance to *Fov* remain unclear. Herein it is reported that *Fov7*, a gene unlike canonical plant disease‐resistance (*R*) genes, putatively encoding a GLUTAMATE RECEPTOR‐LIKE (GLR) protein, confers resistance to *Fov* race 7 in Upland cotton. A single nucleotide polymorphism (SNP) (C/A) in *GhGLR4.8*, resulting in an amino acid change (L/I), is associated with *Fov* resistance. A PCR‐based DNA marker (*GhGLR4.8^SNP(A/C)^*) is developed and shown to cosegregate with the *Fov* resistance. CRISPR/Cas9‐mediated knockout of *Fov7* results in cotton lines extremely susceptible to *Fov* race 7 with a loss of the ability to induce calcium influx in response to total secreted proteins (SEPs) of *Fov*. Furthermore, coinfiltration of SEPs with *GhGLR4.8^A^* results in a hypersensitive response. This first report of a GLR‐encoding gene that functions as an *R* gene provides a new insight into plant–pathogen interactions and a new handle to develop cotton cultivars with resistance to *Fov* race 7.

## Graphical abstract

*Fusarium oxysporum* f. sp. *vasinfectum* (*Fov*) is a widely distributed fungal pathogen seriously threatening cotton production. Herein, a GLUTAMATE RECEPTOR‐LIKE gene (*GhGLR4.8*) is identified as an atypical disease‐resistance gene specifying resistance to *Fov* race 7 through a genome‐wide association study in upland cotton, which provides a new insight into plant–pathogen interactions and facilitates the development of elite *Fov*‐resistant cultivars.

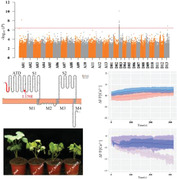

## 1 Introduction

*Fusarium oxysporum* is a soilborne fungal pathogen that infects more than 100 plant species and causes tremendous economic losses in numerous important crop plants, including cotton, tomato, banana, and melons.^[^ ^1^ ^]^ *Fusarium oxysporum* f. sp. *vasinfectum* (*Fov*) causes Fusarium wilt (FW) of cotton, seriously threatening cotton production worldwide.^[^ ^2^, ^3^, ^4^ ^]^ *Fov* is classified into eight races (1–8) based on pathogenicity to different cotton species and other crops.^[^ ^4^ ^]^ In China, three races (races 3, 7, and 8) of *Fov* have been reported, and race 7 is identified as the most widely dispread race and possesses the highest virulence.^[^ ^5^ ^]^ Chlamydospores produced by *Fov* can survive in the soil for long period in the absence of host plants, which makes *Fov* a difficult pathogen to be managed and eliminated in fields.^[^ ^3^ ^]^

Phenotypic and genetic analyses have revealed that one or two major *R* genes with complete to incomplete dominance, together with a few minor genes, contribute to *Fov* resistance. Three major resistance genes (*FW^R^*, *Fov1*, *Fov4*) and some quantitative trait loci (QTL) conferring resistance to *Fov* in different cotton species have been identified.^[^ ^2^, ^3^, ^6^, ^7^, ^8^, ^9^ ^]^ The resistance of Upland cotton to *Fov* race 7 is governed by a single dominant gene, *FW^R^*, and was mapped to chromosome 17 (D03).^[^ ^2^ ^]^ *Fov1* was mapped to chromosome 16 and confers resistance to *Fov* race 1 in *G. barbadense* Pima‐S7 and Pima 3‐79^[^ ^7^ ^]^ while *Fov4* was mapped to chromosome 14 and confers resistance to *Fov* race 4 in *G. barbadense* Pima‐S6.^[^ ^8^ ^]^ *GaGSTF9*, a gene encoding the Phi class of glutathione S‐transferases and located on chromosome A11, may be a target for *Fov* resistance in *G. arboretum*.^[^ ^10^ ^]^ Furthermore, *GaGSTF9* was also identified as a candidate gene for Verticillium wilt resistance.^[^ ^11^ ^]^ However, none of the *Fov*‐resistance genes has been characterized in Upland cotton, which accounts for more than 90% of cotton production worldwide. Whether the genes conferring resistance to *Fov* race 7 in *G. hirsutum* and in *G. arboretum* are the same is unclear.

## 2 Results

### 2.1 Genome‐Wide Association Study (GWAS) Analyses for Fusarium Wilt Resistance in Upland Cotton

Through direct screening from susceptible cultivars in disease nurseries, highly FW‐resistant germplasm has been identified, and has been widely deployed for breeding FW‐resistant Upland cotton.^[^ ^9^ ^]^ To assess the favorable genetic variation during artificial selection over the past decades and finely map *Fov*‐resistance genes, a re‐sequenced population of 290 diverse Upland cotton accessions, collected from China, was employed to screen for *Fov* resistance. Among the population, 222 accessions were genotyped previously.^[^ ^12^ ^]^ The resistance of the cotton population was screened for resistance in a disease nursery and the disease index (DI) to FW was investigated. The DI of the population ranged from 0 to 81.6, with 122 accessions susceptible to FW and 168 accessions resistant to FW (Figure S1 and Table S1, Supporting Information). In total, 2 719 708 high‐quality SNPs were identified, and population structure analysis was performed by using these SNPs. The result shows that the values of Evanno's Δ*K* present an obvious spike at *K* = 2 (Figure S2a, Supporting Information), which suggests that the population can be divided into two subpopulations (Figure S2b, Supporting Information). Principal component analysis (PCA) and neighbor‐joining analysis were also performed to further assess the genetic diversity of our new association panel and showed consistent results (Figure S2c,d, Supporting Information).

Candidate gene association analysis was performed to evaluate the association between the homologous genes of *GaGSTF9* in *G. hirsutum* and *Fov* resistance. No SNPs in an upstream region of *GhGSTF9* were associated with *Fov* resistance in *G. hirsutum*, differing from the result of *GaGSTF9* (Figure S3, Supporting Information),^[^ ^10^ ^]^ which suggested there may be a different mechanism for *Fov* resistance between *G. hirsutum* and *G. arboretum*.

**Figure 1:**
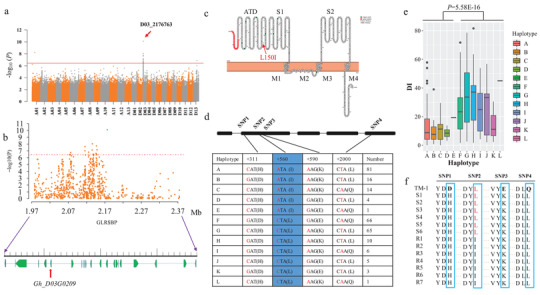
GWAS analysis and identification of natural variation in *GhGLR4.8* associated with Fusarium wilt resistance in cotton. a) Manhattan plot for the Fusarium wilt disease index. The Red solid line represents the Bonferroni‐adjusted significance threshold (−log_10_ (*P*) = 6.43). The most significant SNP (D03_2176763) is marked by the arrowhead. b) Regional Manhattan plot (from 1.97 to 2.37 Mb) for FW resistance on chromosome D03. The annotated genes are indicated by green boxes. c) Predicted structure of *Gh_D03G0209*. Red dots indicate signal peptide sequence. Four transmembrane domains are indicated as M1–M4. Two segments of LBD are indicated as S1 and S2. d) Gene structure display and DNA polymorphisms in the exon of *GhGLR4.8*. Blue‐shared regions indicate the most significant SNP. The numerical value indicates the number of different *GhGLR4.8* haplotypes in 290 accessions. e) Comparison of the disease index (DI) between these haplotypes in the GWAS population. In box plots, center line indicates median, box limits denote upper and lower quartiles, and points indicate outliers. *P*‐value is calculated using two‐tailed Student's *t*‐test. f) Detection of amino acid substitution by four nonsynonymous SNPs in *GhGLR4.8* in FW‐resistant (R1–R7) and FW‐susceptible (S1–S6) cotton cultivars through Sanger sequencing. S1: Emian 11, S2: Esha 28, S3: Ejing 92, S4: Xuzhou 142, S5: Xuzhou 209, S6: Xinluzao 4. R1: Yinshan 4, R2: Zhongmiansuo 12, R3: Jinmian 28, R4: Yumian 19, R5: Xinluzao 31, R6: Xinluzao 36, R7: Xinluzhong 14.

We performed GWAS with the Upland cotton population for *Fov* resistance using a mixed linear model approach.^[^ ^13^ ^]^ The result revealed that a single region on chromosome D03 contains 15 SNPs that are significantly associated with *Fov* resistance, meeting a threshold for Bonferroni correction (*P* < 1/2 719 708, −log_10_ (*P*) > 6.43) (**Figure** **1**a). The most significant SNP D03_2176763 with a −log_10_ (*P)* value of 10.106, accounted for 17.54% of the phenotypic variance (Table S2, Supporting Information). The *Fov* resistance in 194 accessions of this and previous population^[12]^ had been identified through several previous studies and is indicated as HR, R, T, and S (Figure S4a and Table S3, Supporting Information).^[^ ^14^ ^]^ We performed a case‐control association mapping of the 194 accessions, with 127 HR and R accessions as case and 67 T and S accessions as control. A continuous peak was observed on chromosome D03, and D03_2176763 exceeded the significant threshold, consistent with the GWAS result performed in 290 accessions (Figure S4b, Supporting Information). Both assays showed that FW resistance to race 7 in Upland cotton was conferred by a single major locus on chromosome 17 (D03), consistent with a previous study.^[^ ^2^ ^]^ We therefore designated the locus for the resistance of Upland cotton to *Fov* race 7 as *Fov7*.

### 2.2 *GhGLR4.8* Is Significantly Associated with Fusarium Wilt Resistance

FW‐resistance gene in Upland cotton was previously mapped on D03 with an interval genetic distance of 10.8 cM from JESPR304 and 5.7 cM from CIR035.^[^ ^2^ ^]^ Sequence mapping placed the most significant SNP D03_2176763 at a distance of 540 158 and 70 570 bp from JESPR304 and CIR035, respectively (Figure S4c, Supporting Information). Linkage disequilibrium (LD) calculation showed that the LD decay distance for chromosome D03 in this 290 association panel is about 200 kb (Figure S5, Supporting Information), so we estimated a candidate region from 1.97 to 2.37 Mb (200 kb on either side of SNP D03_2176763). A total of 23 putative protein‐encoding genes were found in this 400 kb genomic region (Figure 1b; Table S4, Supporting Information). Although the candidate region on D03 contained 836 polymorphisms, only 17 polymorphisms were significantly associated with FW resistance (Figure 1b). Among them, 2 SNPs resulted in amino acid changes. D03_2125319, which is located in gene *Gh_D03G0206*, resulted in an amino acid change from phenylalanine to serine. *Gh_D03G0206* is an ortholog of the *Arabidopsis CYP83B1* encoding an oxime‐metabolizing enzyme in the glucosinolate biosynthetic pathway, a class of secondary metabolites found mainly in *Brassicaceae*.^[^ ^15^ ^]^ SNP D03_2176763 is located in the second exon of *Gh_D03G0209*, which was annotated as a homologue of the *Arabidopsis* GLUTAMATE RECEPTOR‐LIKE 3.3 (*GLR3.3*)^[^ ^16^ ^]^ playing important roles in the plant innate immune response.^[^ ^17^, ^18^ ^]^ To better understand GLR family members in cotton and possible function of *Gh_D03G0209*, genome‐wide identification and phylogenetic analysis of cotton *GLRs* were performed. A total of 36 genes harboring the essential domains of ionotropic glutamate receptors (iGluRs) were regarded as *bona fide* GLRs through Pfam prediction according to the newly assembled *G. hirsutum* accession Texas Marker‐1 (TM‐1) (Table S5, Supporting Information).^[^ ^19^ ^]^ Phylogenetic analysis revealed that the 36 GLRs in cotton with 20 *A. thaliana* GLRs and three GLRs (OsGLR3.5,^[^ ^20^ ^]^ SlGLR1.1, and SlGLR1.2^[^ ^21^ ^]^) could be divided into four clades (GLR1, GLR2, GLR3, and GLR4) (Figure S6, Supporting Information), Gh_D03G0209 corresponding to Ghir_D03G002390.1 was grouped into clade GLR4 with OsGLR3.5, SlGLR1.1 and SlGLR1.2 (Figure S6, Supporting Information). According to the arrangement of GLR subfamily on chromosome, Gh_D03G0209 was designated as GhGLR4.8. The predicted structure of the protein encoded by *GhGLR4.8* is similar to iGluRs and AtGLRs,^[^ ^22^, ^23^ ^]^ with an extracellular amino‐terminal domain (ATD), two extracellular putative ligand‐banding domains (LBD) (S1 and S2), four transmembrane helices (M1–M4, 1 of which ‐M2‐ is not fully transmembrane), a cytoplasmic tail (carboxylterminal domain; CTD) and a signal peptide at the N terminus (Figure 1c). Sequence alignment of GhGLR4.8, GluA2 and AtGLRs revealed that the SNP D03_2176763 (C/A) lies in the predicted ATD domain causing an amino acid change from leucine (reference) to isoleucine (alternate) (Figure 1c; Figure S7, Supporting Information). Among the 290 members of the association panel, the disease index of accessions carrying the allele ‘AA’ was significantly lower than that of accessions carrying the allele ‘CC’ (Figure S8b, Supporting Information).

Further analysis showed that there are four nonsynonymous SNPs, including the SNP D03_2176763, within the coding region of *Gh_D03G0209* (Figure 1d). Based on these four nonsynonymous SNPs, there are 12 haplotypes for *Gh_D03G0209*., i.e., haplotypes A‐E with the allele ‘AA’ of D03_2176763 and haplotypes F‐L with the allele ‘CC’ of D03_2176763 (Figure 1d). Varieties carrying haplotypes A‐E exhibited significantly lower disease index than haplotypes F‐L (Figure 1e). These results suggest that the two haplotypes based on the lead SNP D03_2176763 are associated with FW‐resistant and FW‐susceptible phenotypes in Upland cotton. Therefore, the gene *Gh_D03G0209* (*GhGLR4.8*) is the most likely candidate gene for *Fov7*.

We then selected 6 highly susceptible and 7 highly resistant Upland cotton cultivars to sequence *GhGLR4.8*. The results revealed that all resistant varieties carry ‘A’ alleles (*GhGLR4.8^A^*) and all susceptible varieties carry ‘C’ alleles (*GhGLR4.8^C^*) at the position 2176763 of chromosome D03 (Figure 1f). The other three variations in *GhGLR4.8* were not linked with *Fov* resistance (Figure S8a,c,d, Supporting Information) and did not differ consistently between resistant and susceptible cultivars (Figure 1f). Surprisingly, we found that no cotton in the wild group (wild cottons) was of the *GhGLR4.8^A^* genotype and a very low percentage of *GhGLR4.8^A^* genotype was identified in cottons from the ABI group (cottons from America, Brazil and India) (Figure S9, Supporting Information). On the contrary, the proportion of *GhGLR4.8^A^* genotype increased to 43% in cultivars from China (Figure S9, Supporting Information), suggesting evolution of *GhGLR4.8* during cotton improvement. These results suggest that the SNP D03_2176763 is responsible for the variation of FW resistance in Upland cotton and *GhGLR4.8^A^* may be the causal resistance gene for *Fov7*.

### 2.3 *GhGLR4.8^A^* Confers Resistance to Fusarium Wilt in Upland Cotton

To investigate whether the expression levels of *GhGLR4.8* are associated with FW resistance in the natural population, 9 lines each of FW‐resistant and FW‐susceptible varieties were selected to determine the expression levels of *GhGLR4.8* by quantitative RT‐PCR (qRT‐PCR). No significant difference (Fold change < 2) was found in transcript level of *GhGLR4.8* between susceptible and resistant varieties with or without *Fov* inoculation (Figure S10, Supporting Information), suggesting that *GhGLR4.8*‐mediated resistance to *Fov* is independent of its expression level. Within the FW resistance locus, there are a total of 23 genes (Table S4, Supporting Information). To determine the possible functions of these genes in FW resistance, virus‐induced gene silencing (VIGS) constructs were completed for all these 23 genes excluding *Gh_D03G0224*, the expression level of which was extremely low so we failed to amplify it. We assigned the rest 22 genes excluding *Gh_D03G0209* to 3 groups with seven genes in each group. VIGS constructs for each gene in the individual group were mixed equally to generate multigene‐silenced cotton plants named *TRV:groupI*, *TRV:groupII*, and *TRV:groupIII*, respectively, according to our previous study.^[^ ^24^ ^]^ The results of qRT‐PCR showed that the expression levels of these genes were successfully knocked down in seedlings 14 days after *Agrobacterium* infiltration (Figure S11b,c,d, Supporting Information). All three *TRV:group* cotton lines showed high resistance to *Fov* similar to the empty‐vector‐carrying cotton lines (*TRV:00*) (Figure S11a, Supporting Information). Meanwhile, *GhGLR4.8* was knocked down in two highly resistant Upland cotton cultivars (YZ1 and Xinluzao 46) and one susceptible Upland cotton cultivar (Xinluzao 7) (Figure S12b, Supporting Information). The results showed that, compared with the *TRV:00* plants, knockdown of *GhGLR4.8^A^* in YZ1 and Xinluzao 46 made the plants severely susceptible to *Fov* race 7, with severe leaf wilting and brown coloration in the vascular tissue and high disease severity (Figure S12a,c,d, Supporting Information). Furthermore, fungal biomass analysis showed that the amount of fungal DNA in *TRV:GhGLR4.8^A^* plants was significantly higher than that in *TRV:00* plants (Figure S12e, Supporting Information). No significant difference in disease symptoms and fungal content was observed when *GhGLR4.8^C^* was knocked down in FW‐susceptible cotton cultivar Xinluzao 7 (Figure S12, Supporting Information). We also found that there was no significant effect on Verticillium wilt resistance when *GhGLR4.8^A^* was knocked down (Figure S13, Supporting Information). Taken together, these results suggested that *GhGLR4.8^A^* is the causal resistance gene for *Fov7* specifying resistance to *Fov* race 7 in Upland cotton.

**Figure 2:**
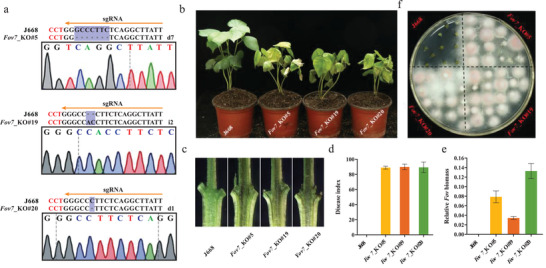
CRISPR/Cas9‐mediated knockout of *Fov7* strongly suppresses resistance to *Fov* in Upland cotton. a) Identification of mutation type in knockout lines by PCR‐based sequencing. Three representative transgenic lines were generated in the J668 genetic background. The designations of d7, i2, and d1 denote a 7 bp deletion, a 2 bp insertion and a 1 bp deletion, respectively. b) Disease symptoms of J668 plants and three knockout transgenic lines at 20 days after inoculation with *Fov*. c) Vascular bundle coloration in longitudinal sections of inoculated J668 and transgenic stem. d) Disease index statistics of J668 and transgenic plants at 3 weeks after *Fov* inoculation. e) Relative content of *Fov* DNA in inoculated stem of J668 and transgenic plants. f) Fungal recovery assay of J668 and three knockout transgenic plants. Short sections cut from inoculated plants were incubated on potato dextrose agar (PDA) medium and the color of *Fov* mycelium is purple‐red. Data in d) and e) are presented as mean ± SD from three biological replicates.

To further validate the role of *Fov7* in resistance to *Fov*, we used CRISPR/Cas9 technology to knock out the *Fov7* gene in Jin668 (J668), a highly resistant cultivar. A 20‐nt sequence in the *Fov7* gene was chosen as the target site for Cas9 cleavage (**Figure** **2**a) and generated multiple putative transgenic lines in the J668 background. The type of mutations were identified by Hi‐TOM.^[^ ^25^ ^]^ Three lines (*Fov7*_KO#5, *Fov7*_KO#19, and *Fov7*_KO#20) were further verified through Sanger sequencing. The results showed seven‐base and single‐base deletions in *Fov7* in *Fov7*_KO#5 and *Fov7*_KO#20 lines, respectively, while a two‐base insertion was observed in *Fov7*_KO#19 line, leading to a frameshift mutation (Figure 2a). After inoculation with *Fov* race 7, all three *Fov7_*KO lines exhibited extremely severe wilt symptoms, with obvious brown coloration of the vascular tissue, a high disease index and high levels of fungal DNA. In contrast, wild type plants were highly resistant to *Fov*, almost no brown coloration was observed and no fungal DNA was detected (Figure 2b–e). Additionally, *Fov* hyphae recovered from infected *Fov7_*KO lines grew well in culture, while no obvious *Fov* hyphae were recovered from infected wild type plants (Figure 2f). These results indicate that knocking out of *Fov7* resulted in a loss of resistance to *Fov* (Figure 2).

### 2.4 Cosegregation between *GhGLR4.8^SNP(A/C)^* Marker and Fusarium Wilt Resistance

**Figure 3:**
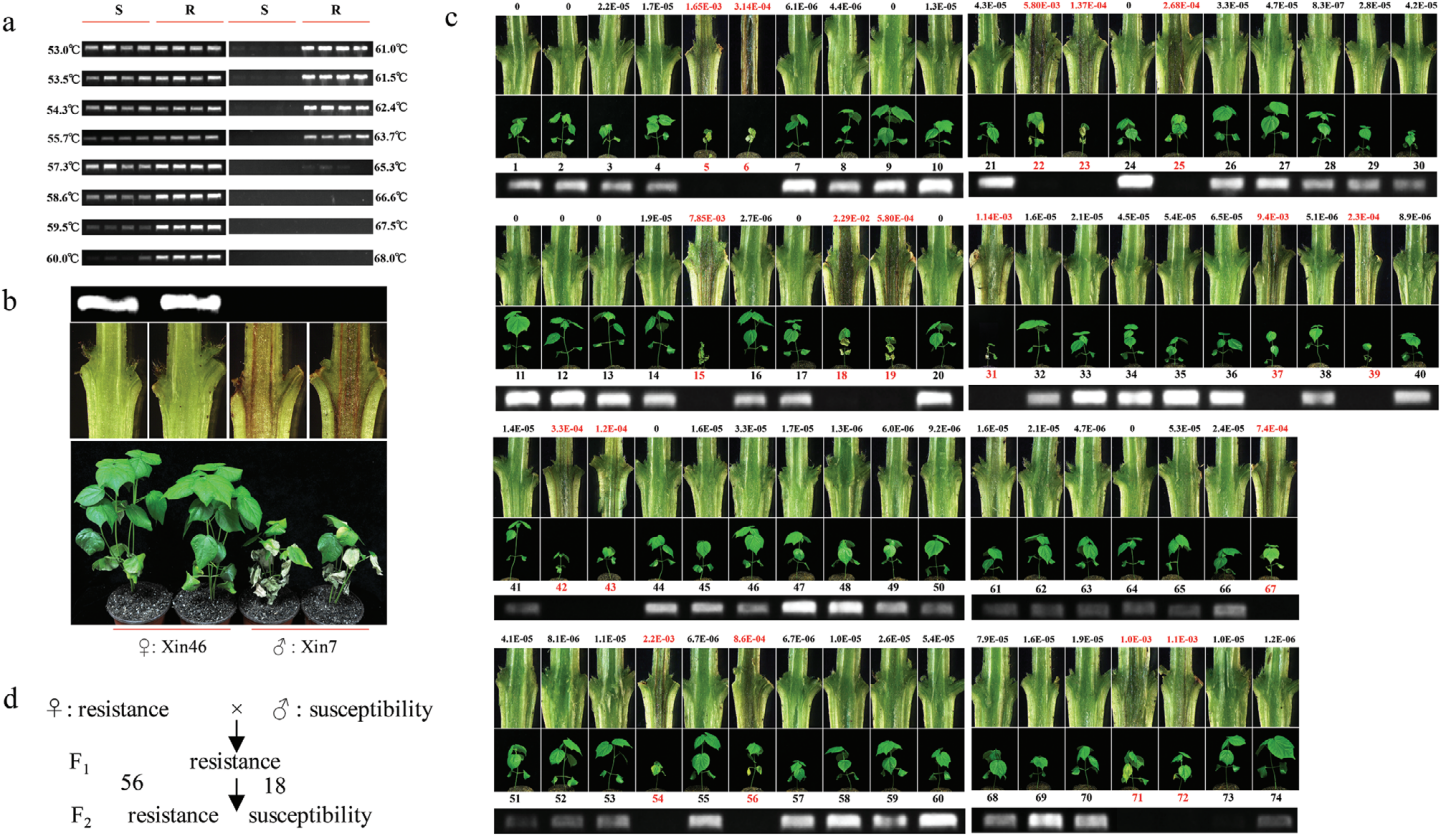
Cosegregation between the *GhGLR4.8^SNP(A/C)^*marker and FW resistance. a) Development of a PCR‐based DNA marker based on the nucleotide variation in *GhGLR4.8* between resistant (R) and susceptible (S) cotton. Four susceptible cotton cultivars (Ejing 1, Jimian 8, Xinluzao 8, Emian11) and four resistant cotton cultivars (Yinshan 4, Zhongmiansuo 12, Jinmian 28, Yumian 11) were selected to explore suitable annealing temperature. Under annealing temperature of 61–63.7 °C, *GhGLR4.8^A^* and *GhGLR4.8^C^* genotype were distinguished based on the presence of a diagnostic band. b) Phenotype and genotype of Xinluzao 46 (Xin46) and Xinluzao 7 (Xin7). Xin46 served as female parent line and Xin7 serve as male line. c) Phenotype and genotype of F_2_ population derived from Xin46 × Xin7 cross. The resistance of F_2_ plants to *Fov* was reflected by disease symptom, vascular bundle coloration and the relative content of fungal DNA (indicated by values above the dissected stem). The numbers in the middle of seedlings and PCR band indicate individual F_2_ plants. The numbers and values of susceptible F_2_ individuals are marked in red. Resistance/susceptibility phenotype corresponded to the presence/absence of PCR bands. d) Calculation of the segregation ratio of resistant plants to susceptible plants (56R:18S, *χ* ^2^ = 0.037).

Based on the observation that resistant varieties carry the allele *GhGLR4.8^A^* and susceptible varieties carry the allele *GhGLR4.8^C^*, we designed a pair of primers with the polymorphism located at 3’ end of the forward primer. A gradient annealing temperature PCR was employed to find the optimal annealing temperature which can distinguish the DNA polymorphisms in *GhGLR4.8* between resistant and susceptible varieties. Gel electrophoresis indicated that an obvious product with a size of 464 bp could be successfully amplified for *GhGLR4.8^A^* in four resistant varieties, whereas no clear product was detected for *GhGLR4.8^C^* in four susceptible varieties at the optimal annealing temperature (61–63.7 °C) (**Figure** **3**a).

To further verify *GhGLR4.8^A^* is responsible for *Fov* resistance in cotton, an F_2_ segregation population was generated from a cross between the highly resistant cultivar Xinluzao 46 and the susceptible cultivar Xinluzao 7. First, the biparental lines were genotyped using the *GhGLR4.8^SNP(A/C)^* marker and phenotyped after inoculation with *Fov*. Xinluzao 7 was genotyped as *GhGLR4.8^C^*, and Xinluzao 46 was genotyped as *GhGLR4.8^A^* (Figure 3b). Then the F_2_ population comprising 74 individual plants were genotyped using the *GhGLR4.8^SNP(A/C)^* marker and the resistance to *Fov* was evaluated through pathogenicity tests. Among the 74 individual plants tested, 56 lines were genotyped as *GhGLR4.8^A^* and 18 lines were genotyped as *GhGLR4.8^C^* (Figure 3c). All the individuals genotyped as *GhGLR4.8^A^* were robust, exhibiting almost no brown coloration in the vascular tissues and an extremely low content of *Fov* in the stem after inoculation. While all the individuals genotyped as *GhGLR4.8^C^* exhibited severe leaf wilting, brown coloration in vascular tissues and a high content of *Fov* in the stem (Figure 3c). Based on the molecular identification and inoculation response, the segregation ratio of resistant plants to susceptible plants fit a 3:1 ratio (56R:18S, *χ* ^2^ = 0.037) (Figure 3d). A repeated experiment was conducted and similar results were obtained (Figure S14, Supporting Information). These results strongly implicate *GhGLR4.8^A^* as the causal resistance gene for *Fov7*, conferring resistance to *Fov* race 7 in Upland cotton.

### 2.5 *GhGLR4.8^A^* Triggers Immune Response and Induces Calcium Influx in Response to *Fov*

**Figure 4:**
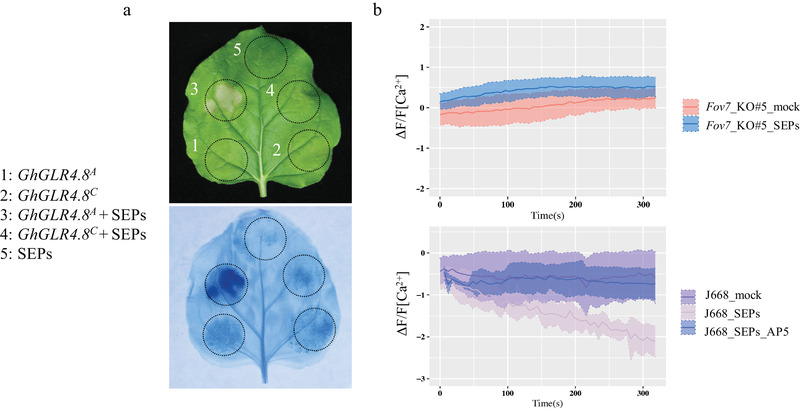
Total secreted proteins of *Fov* activate Ca^2+^ influx and trigger a hypersensitive response. a) Analysis of hypersensitive response triggered by coinfiltration of *GhGLR4.8* and total *Fov* secreted proteins (SEPs) in *Nicotiana benthamiana*. *GhGLR4.8^A^* and *GhGLR4.8^C^* were expressed by *Agrobacterium*‐mediated transient transformation (Agro‐infiltration). For coinfiltration, tobacco leaves were infiltrated with SEPs 48 h after Agro‐infiltration. Cell death triggered by coinfiltration was visualized by trypan blue staining 60 h after Agro‐infiltration. Representative photographs are shown. b) Measurements of Ca^2+^ influx triggered by SEPs in cotton root meristem of *Fov7* knockout and J668 seedlings by the scanning ion‐selective electrode method. AP5, an iGluRs antagonist. Error bars, mean ± SD, *n* = 4.

To examine whether *Fov7* as a FW‐resistance gene could trigger plant immune response in responses to *Fov*, we performed *Agrobacterium tumefaciens* infiltration to transiently express *GhGLR4.8^A^* and *GhGLR4.8^C^* in *Nicotiana benthamiana*. Total secreted proteins (SEPs) of *Fov* were isolated and infiltrated in *N. benthamiana* 48 h after *Agrobacterium* infiltration. The results showed that coinfiltration of SEPs with *GhGLR4.8^A^*, but not with *GhGLR4.8^C^*, resulted in a hypersensitive response, which indicates that *Fov* could be recognized by *GhGLR4.8^A^* to trigger the plant immune response (**Figure** **4**a).

The initiation of innate immunity responses upon specific microbial epitopes, like flg22, elf18, chitin, and also wound signaling recognition, involve an apoplastic Ca^2+^ influx via GLR channels.^[^ ^26^, ^27^ ^]^ To verify the function of *GhGLR4.8* in the regulation of Ca^2+^ influx following application of the SEPs of *Fov*, we performed scanning ion‐selective electrode assays to measure the calcium ion flux of cotton roots treated with SEPs. The results showed that the SEPs of *Fov* induced an increase in averaged Ca^2+^ influx in J668 carrying *GhGLR4.8^A^*. The iGluRs antagonist (2R)‐amino‐5‐phosphonopentanoate (AP5) significantly suppressed the SEPs‐induced response. The increase of [Ca^2+^]_cyt_ influx observed in the *GhGLR4.8^A^* roots induced by SEPs was completely absent in the roots of *Fov7‐*knockout plants (Figure 4b).

### 2.6 Knockout of *GhGLR4.8* Impairs Cotton Cell Wall Fortification in Response to *Fov*

To identify the signaling pathways regulated by *GhGLR4.8* in response to pathogen, a dual RNA sequencing (RNA‐seq) was carried out to analyze the transcriptome profile of both plant and pathogen genes in *Fov*‐infected hypocotyls of J668 and *Fov7*_KO#5 at 5 days after inoculation (dpi) and 10 dpi. The trimmed reads from samples of *Fov*‐infected *Fov7*_KO#5 hypocotyls and in vitro‐grown *Fov* were mapped to the genome of *Fov* race 4.^[^ ^28^ ^]^ It was found that 0.08–0.68% of the total reads derived from *Fov* at 5–10 dpi, yielding uniquely aligned read counts from 20799 to 236807 (Table S6, Supporting Information). The varied fungal mRNA reads over the time course revealed hyphal proliferation within host xylem vessels. Of all the differentially expressed fungal genes (DEFGs), 1297 and 2759 host‐induced genes were identified at 5 dpi and 10 dpi, respectively (Figure S15a,b; Table S7, Supporting Information). Gene ontology (GO) enrichment analysis showed that the categories of carbohydrate metabolic process was highly enriched among host‐induced genes, most of which (70/93) were glycoside hydrolase (Figure S15c and Table S7, Supporting Information), one of the families of cell wall‐degrading enzymes (CWDEs).

**Figure 5:**
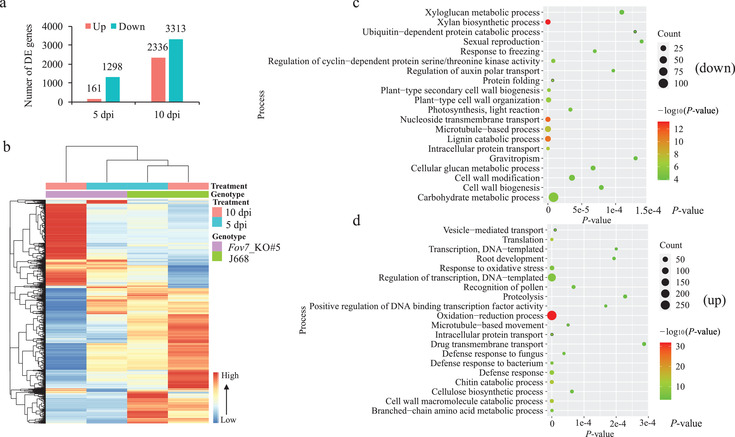
Transcriptome profiles of genes in hypocotyls of J668 and *Fov7*_KO#5 at various time points after infection with *Fov*. a) Number of differentially expressed plant genes (DEPGs) (*P* < 0.05, |log_2_(FC)| > 2) in hypocotyls of *Fov*‐infected *Fov7*_KO#5 versus J668 at different time points after inoculation. Fold change is calculated by inoculated *Fov7*_KO#5/J668. dpi, days post inoculation. b) Heat map of DEPGs in hypocotyls of *Fov7*_KO#5 versus J668 at different time points after inoculation. c,d) Gene ontology (GO) enrichment analysis of all c) down‐regulated or all d) up‐regulated genes. TOP 20 significantly enriched biological process GO terms are show. Three biological replicates were included for each treatment.

Of all the differentially expressed plant genes (DEPGs) in hypocotyls of *Fov7*_KO#5 compared with J668, 161 and 2336 genes were upregulated, while 1298 and 3313 genes were downregulated at 5 dpi and 10 dpi, respectively (**Figure** **5**a,b; Table S8, Supporting Information). More DEPGs were also identified in *Fov7*_KO#5 than that in J668 at 10 dpi versus 5 dpi (Figure S16a,b and Table S9, Supporting Information), highlighting a stronger response in *Fov7*_KO#5 to *Fov* versus J668. Consistent with the increased secretion of glycoside hydrolases during *Fov* colonization, host genes involved in cell wall fortification were highly enriched among downregulated genes, revealing that knockout of *GhGLR4.8^A^* impaired the process of cell wall fortification in response to *Fov* (Figure 5c; Table S8, Supporting Information). Surprisingly, genes related to plant defense were highly enriched among upregulated genes (Figure 5d; Table S8, Supporting Information). Moreover, enhanced plant defense‐related responses and reduced cell wall fortifications were observed in *Fov7*_KO#5 at 10 dpi versus 5 dpi (Figure S16c and Table S9, Supporting Information), while weakened plant defense‐related responses were observed in J668 at 10 dpi versus 5 dpi (Figure S16d and Table S9, Supporting Information). These results suggests that loss‐of‐function of *GhGLR4.8^A^* impairs cell wall fortification during cotton‐*Fov* interaction and defense‐related response is enhanced by more colonization of *Fov* in *Fov7*_KO#5 xylem vessels.

## 3 Discussion

Employment of disease‐resistance genes are considered as one of the most efficient strategies to control plant disease.^[^ ^29^ ^]^ Significant achievement has been made in breeding for FW resistance through traditional phenotype evaluation under field conditions. However, the evolution of new and highly virulent *Fov* races have emerged as one of major threats to cotton production.^[^ ^9^ ^]^ The identification and characterization of disease resistance genes is indispensable for both understanding resistance mechanism and efficient crop genetic improvement.

Herein, we identified the FW‐resistance gene, *Fov7*, conferring resistance against *Fov* race 7 in Upland cotton and developed a PCR‐based DNA markers associated with *Fov* resistance. *Fov7* is located on chromosome 17 (D03), consistent with previous molecular mapping with F_2_ populations^[^ ^2^, ^30^ ^]^ but different from the chromosomal location observed in *G. arboretum*.^[^ ^10^ ^]^ Different location of QTLs for *Fov* resistance among interspecific and intraspecific populations suggested a different genetic basis of *Fov* resistance in *G. hirsutum* and *G. barbadense*.^[^ ^30^ ^]^ Our results showed that *Fov7* varies greatly in different species, especially in *G. barbadense* (Figure S17, Supporting Information). Unlike *G. hirsutum*, most *G. barbadense* cultivars are susceptible to *Fov* race 7 in China.^[^ ^31^ ^]^ Thus, *Fov7* can be employed to *G. barbadense* breeding through marker assisted selection.

*Fov7* encodes a GLUTAMATE RECEPTOR‐LIKE (GLR) protein, which is distinct from typical race‐specific disease resistance proteins. The tomato‐*Fol* pathosystem is a well‐established model system to study plant‐*F. oxysporum* interactions.^[^ ^32^ ^]^ *R* genes in tomato conferring resistance to different *Fol* races have been characterized.^[^ ^32^, ^33^, ^34^, ^35^ ^]^ The I gene for *Fol* race 1 encodes a membrane anchored leucine‐rich repeat receptor‐like protein (LRR‐RLP);^[^ ^35^ ^]^ the I‐2 gene for *Fol* race 2 encodes a coiled‐coil nucleotide‐binding leucine‐rich repeat (CC‐NB‐LRR) protein.^[^ ^36^ ^]^ The I‐3 and I‐7 genes for *Fol* race 3 encode an S‐receptor‐like kinase (SRLK)^[^ ^37^ ^]^ and leucine‐rich repeat receptor‐like protein (LRR‐RLP),^[^ ^32^ ^]^ respectively. In our study, *GhGLR4.8^A^* was identified as a major *R* gene specifying resistance to *Fov* in Upland cotton, suggesting the molecular basis of cotton against *Fov* was distinct from tomato against *Fol*, which provides a new insight into plant‐*F. oxysporum* interactions. An increasing number of atypical resistance genes have been identified. Examples include *Lr34* and *Lr67* against wheat rust diseases,^[^ ^38^, ^39^ ^]^ *Fhb1* and *Fhb7* against Fusarium head blight in wheat,^[^ ^40^, ^41^ ^]^ and *ZmFBL41* against banded leaf and sheath blight in maize.^[^ ^42^ ^]^ Like *ZmFBL41*, the expression of *GhGLR4.8* showed no difference between resistant and susceptible cotton cultivars (Figure S10, Supporting Information). Most of the known atypical disease resistance genes confer resistance to multiple pathogens. However, *Fov7* just confers resistance to *Fov* but not to *V. dahliae* (Figure S13, Supporting Information), which causes another disastrous fungal disease that threatens cotton production worldwide.

Glutamate receptors are best known for their role as neurotransmitters mediating excitatory synaptic transmission in vertebrate brain.^[^ ^43^ ^]^ Most recently, GLR‐3 was identified as a cold receptor in the peripheral sensory neuron ASER, suggesting the multifunction of GLRs acting as both chemical receptor and thermal receptor.^[^ ^44^ ^]^ Plant GLRs are homologues of mammalian iGluRs^[^ ^45^ ^]^ and have evolved many plant‐specific physiological functions, such as in sperm chemotaxis and transcriptional regulation,^[^ ^46^ ^]^ pollen tube morphogenesis,^[^ ^47^ ^]^ leaf‐to‐leaf wound signaling,^[^ ^27^, ^48^ ^]^ and in the plant defense response.^[^ ^17^, ^18^ ^]^ A total of 20 GLRs have been identified in *A. thaliana* and were grouped into three clades.^[^ ^49^ ^]^ Moreover, a fourth clade of GLRs has been found in some monocotyledons and dicotyledons.^[^ ^50^, ^51^ ^]^ Phylogenetic analysis showed that cotton GLRs were divided into four subfamilies and GhGLR4.8 was grouped into clade GLR4 (Figure S6, Supporting Information), suggesting evolutionary expansion of *GLR* gene family in cotton. GLR3 subfamily was the most studied plant GLRs, *AtGLR3.4* and the related *AtGLR3.2* were primarily expressed in the phloem of roots,^[^ ^52^ ^]^ *AtGLR3.3* and *AtGLR3.6* were localized to the phloem and xylem parenchyma in leaves, respectively,^[^ ^18^ ^]^ and pear *GLR4* was preferentially expressed in phloem.^[^ ^51^ ^]^ *GhGLR4.8^A^* was identified as *Fov7* mediating the resistance to *Fov*, a xylem‐colonizing fungus, suggesting that acquired resistance to *Fov* might be related to a different response between *GhGLR4.8^A^* and *GhGLR4.8^C^* vascular cells. The plant cell wall serves as both mechanical and defensive barrier to restrict the invading of pathogens, alterations of plant cell wall have been demonstrated to have a significant impact on disease resistance.^[^ ^53^ ^]^ To gain access to the cell of plants, pathogens produce an array of cell wall‐degrading enzymes (CWDEs) to break down the barrier.^[^ ^54^ ^]^ Our results showed that knockout of *GhGLR4.8^A^* impaired the process of cell wall fortification in response to *Fov* (Figure 5c; Table S8, Supporting Information), consistent with the highly enriched categories of carbohydrate metabolic process among host‐induced genes (Figure S15c and Table S7, Supporting Information).

Previous studies suggested GLRs act as amino acid‐gated Ca^2+^ channels to perceive changes in apoplastic amino acid concentrations in the regulation of plant defense responses.^[^ ^17^, ^18^ ^]^ Thus far, 12 proteinogenic amino acids, and also GSH, have been identified as GLRs agonists.^[^ ^17^ ^]^ Moreover, Glu is indicated as a new DAMP (damage‐associated molecular pattern) activating GLR ion channels, eliciting defense signal propagation through altered [Ca^2+^]_cyt_.^[^ ^18^ ^]^ Pull‐down experiments suggested that *Fol* Six4 (Avr1) can interact with glutamate decarboxylase,^[^ ^34^ ^]^ an enzyme catalyze glutamate to gamma‐aminobutyric acid. We showed that SEPs of *Fov* could activate Ca^2+^ influx and trigger a hypersensitive response in a GLR‐dependent manner (Figure 4), suggesting that *Fov7* may directly or indirectly interact with *Fov* effectors. There is evidence that some atypical resistance proteins could not only bind small molecules but also bind proteinaceous ligands.^[^ ^55^ ^]^ For example, WAKs respond to changes during pathogen attack through binding with oligogalacturonides, which are regarded as a DAMP.^[^ ^56^, ^57^ ^]^ Recently, a study reported that the wheat *Stb6* gene, encoding a conserved wall associated receptor kinase (WAK)‐like protein, detects the presence of its matching Avr effectors (AvrStb6) to control qualitative pathogen resistance in a gene‐for‐gene manner.^[^ ^55^ ^]^ The direct or indirect interaction between *Fov7* and the corresponding effectors of *Fov* race 7 requires further exploration.

## 4 Conclusions

In the present study, an atypical disease‐resistance gene specifying resistance to *Fov* race 7 was identified in Upland cotton through integrating a genome‐wide association study with gene function analyses. We found that a point mutation in the exon of *GhGLR4.8* was associated with field‐evolved resistance of Upland cotton to Fusarium wilt. CRISPR/Cas9 editing of the gene validated its function and a PCR‐based DNA marker for this polymorphism was developed and subsequently shown to cosegregate with *Fov* resistance in an F_2_ segregation population. Furthermore, the resistant genotype of *GhGLR4.8* (*GhGLR4.8^A^*) triggered immune response and induced calcium ion influx in response to SEPs of *Fov*, indicating that some unknown *Fov* secreted protein has the potential to activate GLR‐mediated ion channel, and RNA‐seq analyses revealed that knockout of *GhGLR4.8^A^* impaired cotton cell wall fortification in response to *Fov*. We demonstrate that *GhGLR4.8* acts as an atypical *R* gene specifying resistance to *Fov* race 7, providing new insights into the interaction between plants and *F. oxysporum*, and the basis of *GhGLR4.8* marker‐assisted selection to develop elite Fusarium wilt‐resistant cultivars.

## 5 Experimental Section

##### Plant Materials and Field Assays

The cotton varieties used in this study were inbred cultivars of Upland cotton and derived from China. A total of 290 cotton accessions were collected to perform GWAS for Fusarium wilt resistance, of which 222 accessions were selected from the previously described 352 resequenced accessions.^[^ ^12^ ^]^ The population was grown in a heavily *Fov*‐infected fields at Kuche, Xinjiang, China. The F_2_ population resulted from the cross Xinluzao 46 × Xinluzao7, two cultivars widely planted in Xinjiang province. Xinluzao 46 is a highly resistant cultivar and Xinluzao7 exhibits susceptibly to *Fov*. The cross and subsequent self‐pollinated of F_1_ were both conducted in greenhouse at Huazhong Agricultural University.

The disease severity of the cotton populations was scored by the Fusarium wilt disease grade (0, 1, 2, 3, 4) and disease index (DI). Evaluation of disease grade and DI followed the technical specifications for evaluating resistance of cotton to diseases and insect pests‐part 4: Fusarium wilt (GB/T 22101.4‐2009). The resistance of cotton were classified into five levels based on the relative disease index (RDI), where RDI = 0 indicates immunity (I), 0 < RDI ≤ 5 indicates high resistance (HR), 5 < RDI ≤ 10 indicates resistance (R), 10 < RDI ≤ 20 indicates tolerance (T) and RDI > 20 indicates susceptibly (S). The resistance levels of the 352 cotton accessions were also searched from a monographs named ‘cotton varieties and genealogy in China’,^[^ ^14^ ^]^ and found that a number of 194 cultivars have been identified through national varieties certification under fields to acquire its resistance. In total, there were 53 HR varieties, 74 R varieties, 22 T varieties, and 45 S varieties.

##### Variation Calling, Population Structure and Linkage Disequilibrium (LD) Analysis

Paired‐end resequencing reads were mapped to the TM‐1 genome^[16]^ and SNP calling were performed as previously described.^[12]^ STRUCTURE (version 2.3.4) was used to analyze the population structure of the 290 cotton accessions.^[^ ^58^ ^]^ TASSEL (version 5.0) was used to perform PCA.^[^ ^59^ ^]^ LD was calculated by Plink (version 1.07) software.^[^ ^60^ ^]^

##### Genome‐Wide Association Studies for Fusarium Wilt Resistance

A total of 2 719 708 high‐quality SNPs (MAF > 0.05) were used to perform GWAS for Fusarium wilt resistance measured by DI in 290 accessions. The compressed mixed linear model (P + G + Q + K) of TASSEL (version 5.0) was used to perform association analysis.^[^ ^13^, ^59^ ^]^ The threshold of significant association was set as *P* = 1/*N* (*N* indicates the number of SNPs). For the 194‐accessions panel, Fusarium wilt resistance was measured by resistance grade. A total of 2 143 700 high‐quality SNPs (MAF > 0.05) were used to perform a case‐control association using Plink (version 1.07) software.^[^ ^60^ ^]^ HR and R were taken as case, T and S were taken as control.

##### Identification of GLR Gene Family Members in Upland Cotton

Hidden Markov model (HMM) was used to identify cotton *GLR* candidate genes. The protein sequence of TM‐1 genome^[19]^ was download from cottongen (https://www.cottongen.org), and the HMM files of PF00060 (ligand‐gated ion channel) and PF00497 (solute binding protein) were download from Pfam database (http://pfam.xfam.org). Hmmer3.2 was used to search the protein sequence to identify *GLR* candidate genes,^[^ ^61^ ^]^ and the genes with PF00060 and PF00497 domains were considered as bona fide GLRs.

##### Phylogenetic Analysis, Structure Predication, and Sequence Alignment

Construction of phylogenetic tree was performed using the neighborjoining (NJ) method using MEGA6.0 software.^[^ ^62^ ^]^ Protein feature visualization was performed using Protter software,^[^ ^63^ ^]^ and transmembrane predictions were performed using TMHMM. Protein sequence alignments were performed with DNAMAN software (Lynnon Biosoft). The figures of alignments were prepared with ESPript (http://espript.ibcp.fr). Amino acids sequences were obtained from The *Arabidopsis* Information Resource (https://www.arabidopsis.org) for ATGLRs, NCBI Protein database (https://www.ncbi.nlm.nih.gov/protein) for GluA2 and cottongen for GhGLRs. The LBD boundaries for GhGLR4.8 were predicted using the alignment of GhGLR4.8 and AtGLRs as guidelines. The ATD boundaries for GhGLR4.8 were predicted using the alignment of GhGLR4.8 and GluA2 as guidelines.

##### *Fov* Inoculation

*Fov* race 7 isolate F17 was cultured in potato lactose broth (PLB, 200 g potato and 20 g lactose per 1 liter) for 3–4 days. The concentration of spores was adjusted to 1 × 10^7^ conidia per mL for inoculation. Cotton seedlings prepared to inoculate were cultivated in Hoagland's solution in a controlled environment chamber under a 16 h light/8 h dark cycle at 25 °C. Roots of the prepared cotton plants were dipped into the spore suspension for 30 min then transplanted into sterilized soil for growing and observing the symptom of Fusarium wilt.

##### Qualitative and Quantitative Detection of *Fov* in Stem of Infected Cotton Plants

Qualitative detection of *Fov* was monitored by cotton dissection and a fungal recovery assay. The fungal recovery assay followed previous methods described by Fradin.^[^ ^64^ ^]^ Briefly, first internode of seedlings was cutoff, after surface sterilized, cut them into short slices and incubated on PDA solid medium at 25 °C. Fungal colonization was evaluated by brown coloration in vascular tissue and DNA content of *Fov* in stem at 20 days after inoculation. Cotyledon nodes were dissected to longitudinal cross‐section and the brown coloration was observed under a stereoscopic microscope (MZFLIII; Leica, Wetzlar, Germany). Quantitative detection of *Fov* in cotton was conducted by qRT‐PCR. The amplification of the *Fov* specific gene was compared to that of the cotton *UB7* to quantify fungal DNA levels according to the method of Abd‐Elsalam et al.^[^ ^65^ ^]^

##### VIGS Analysis

TRV vector construct and *Agrobacterium tumefaciens* infiltration were conducted as previously described but some modifications.^[^ ^66^ ^]^ A 300–500 bp length specific coding sequence of *GhGLR4.8* and other 22 genes were selected as targets for insertion into the TRV:00 vector then amplified from the cDNA of *G. hirsutum* Xinluzao 46. Primer pairs are listed in Table S10 in the Supporting Information. Two restriction endonucleases, *Bam*HI and *Kpn*I, were used to digest TRV:00 plasmid. After purification, the PCR products were fused to the linearized vectors through In‐Fusion Enzyme (Clonetch). The constructs were transformed into *A. tumefaciens* GV3101 by electroporation. For suppression of only one gene, *TRV:GhGLR4.8* Agro‐infiltration was prepared as described by Gao et al.^[^ ^66^ ^]^ For cosuppression of two or more genes together, TRV constructs of the other 21 genes were classified into three groups, and each group comprised 7 TRV constructs which were mixed in equal volumes for Agro‐infiltration according to Miao et al.^[^ ^24^ ^]^ These TRV vectors were then Agro‐infiltrated into the cotyledons of ten‐day‐old seedlings of *G. hirsutum* cv. Xinluzao 46, *G. hirsutum* cv. Xinluzao 7 and *G. hirsutum* cv. YZ1 as described by Gao et al.^[^ ^66^ ^]^ Infiltrated seedlings were grown at 25 °C in a controlled environment chamber with a 16 h light/8 h dark photoperiod cycle. About two weeks after infiltration, qRT‐PCR was performed to detect the expression of each of these genes in leaves to evaluate whether these genes were efficiently knocked down. The primers used for qRT‐PCR are listed in Table S10 in the Supporting Information.

##### CRISPR/Cas9 Gene Editing and Cotton Transformation

CRISPR/Cas9‐mediated knockout of cotton *GhGLR4.8* were carried out as previously described.^[^ ^67^ ^]^ A 20 bp specific targeting sequences for *GhGLR4.8* named sgRNA1 was designed based on homology searches against the cotton genome.^[^ ^16^ ^]^ A fused gRNA‐tRNA‐sgRNA1 was generated using pGTR plasmid as template. pRGEB32‐GhU6.9, a modified vector using cotton endogenous promoter U6.9 to induce the transcription of gRNA, was digested by Endonuclease *Bsa*I. The fused plasmid containing sgRNA1 was ligated to linear pRGEB32‐GhU6.9 by ClonExpress II One Step Cloning Kit (Vazyme), generating *GhGLR4.8* CRISPR/Cas9 constructs. The CRISPR/Cas9 plasmid was transformed into *A. tumefaciens* strain GV3101. Hypocotyls of cotton cultivar *G. hirsutum* cv. J668 were used as explants for genetic transformation of cotton according to previous research.^[^ ^68^ ^]^ After obtaining regenerated plants, the leaf DNA of these transformants was extracted and taken as template for PCR amplification using a primer flanking the target site. The PCR products were sequenced to detect the targeted mutation.

##### Extraction of Total Secreted Protein

Extraction of *Fov* secreted proteins were performed as reported for *Verticillium dahliae* with modification.^[^ ^69^ ^]^ The seedlings of Xinluzao 7 were grown in sterilized MS medium. The *Fov* strain F17 was cultured at 25 °C for 4 days in Czapek liquid medium supplemented with the root sections of seven‐day‐old Xinluzao 7 seedlings. The culture was initially filtered by double gauze then centrifuged at 5000 *g* for 15 min. The culture supernatants were further filtered by passage through a 0.22‐µm filter (Millipore Express PES Membrane). To obtain a high concentration of total secreted proteins, the fungal filtrate was further passed through an Amicon Ultra‐15 3 kDa Centrifugal Filter Devices (Millipore). The volume was concentrated to 1 mL, 14 mL 1× PBS was added and centrifuged again and repeated three times, converting the solution buffer of secreted proteins from Czapek liquid medium to 1× PBS. Finally, the filtrate was concentrated to 500 µL.

##### Agrobacterium‐Mediated Gene Expression in *N. benthamiana*

The DNA sequences of *GhGLR4.8^A^* and *GhGLR4.8^C^* were amplified from Yinshan 4 and Xinluzao 8, respectively, and cloned into pGWB417 using the Gateway system. Transient expression in *N. benthamiana* leaves was performed as previously described.^[^ ^70^ ^]^ *A. tumefaciens* GV3101 cultures containing the *GhGLR4.8* gene were infiltrated at an OD_600_ of 0.2. Total secreted proteins (SEPs) of *Fov* were infiltrated in *N. benthamiana* 48 h after *Agrobacterium* infiltration. SEPs used here was not concentrated through ultra‐filtration, but was the culture supernatants of *Fov* cultured at 25 °C for 4 days in potato lactose broth (PLB, 200 g potato and 20 g lactose per 1 liter) supplemented with the root sections of seven‐day‐old Xinluzao 7 seedlings. Trypan blue was used to stain leaves for cell death 60 h after *Agrobacterium* infiltration. Experiments were replicated biologically three times with five plants each repeat.

##### Measurement of Extracellular Ca^2+^ Influx

Seeds of J668 and one transgenic line (*Fov7*_KO#5) were sterilized and planted in MS medium. One week later, the seedlings were gently removed and transferred into Hoagland's solution one day prior the measurement. Roots were cutoff and balanced in 5 mL testing buffer for 30 min (0.1 × 10 ^−3^ m KCl, 0.1 × 10 ^−3^ m CaCl_2_, 0.1 × 10 ^−3^ m MgCl_2_, 0.5 × 10 ^−3^ m NaCl, 0.3 × 10 ^−3^ m MES, 0.2 × 10 ^−3^ m Na_2_SO_4_, pH 6.0). Then, The Ca^2+^ flux in the meristem of the roots was measured by Xu‐Yue Science & Technology Co. (www.xuyue.net) using Non‐invasive Micro‐test Technology (NMT) as described previously.^[^ ^71^ ^]^ There were at least four seedlings for each treatment. Pharmacological treatments were done by adding 200 µL 1× PBS or concentrated *Fov* total secreted proteins (SEPs) to testing buffer and gently pipetting the solution. Addition of 1× PBS was set as control. J668 seedlings were treated with an iGluRs antagonists (AP 50 µm) 1 h before the SEPs treatment. After 1× PBS and SEPs were added, the Ca^2+^ flux was measured immediately. Ion influx (pmol cm^−2^ s^−1^) was calculated by fractional flux changes (Δ*F*/*F*) using the equation Δ*F*/*F* = (*F* − *F*_0_)/*F*_0_ to correct background intensity values,^[^ ^72^ ^]^ where *F*_0_ denotes the average ion flux of baseline and *F* denotes the ion flux at every 6 s interval.

##### RNA‐Seq Analysis

For RNA‐seq, two‐week‐old seedlings of J668 and *Fov7*_KO#5 were inoculated with *Fov* and hypocotyls were harvested in a time course at 5, 10 d post inoculation (dpi). 10 plants were pooled at each time points. *Fov* was cultured in PLB for 4 days, then the spores were collected. Three biological replicates were included for each treatment. RNA was extracted using PureLink RNA Mini Kit (ambion) from three biological replicates of samples collected at each time point of *Fov*‐inoculated J668 or *Fov7*_KO#5 and from *Fov* spores. Library preparation and Illumina sequencing was performed at Novogene from three biological repeats of samples. Raw reads were trimmed by fastp to get clean reads.^[^ ^73^ ^]^ The trimmed reads of J668 and *Fov7*_KO#5 were uniquely mapped to the TM‐1 genome^[^ ^74^ ^]^ with HISAT2^[^ ^75^ ^]^ using the –dta option, the trimmed reads of *Fov7*_KO#5 were also uniquely mapped to the *Fov* race 4 genome^[^ ^28^ ^]^ with *Fov* spores cultured in vitro as control. Since lacking of gene prediction and annotation of *Fov* race 4 genome, MAKER pipeline^[^ ^76^ ^]^ was used to predict protein‐coding genes of *Fov* race 4, protein domains and Gene Ontology (GO) terms for each gene were annotated using InterProScan.^[^ ^77^ ^]^ Read counts for each gene model was counted using Stringtie and a Python script (http://ccb.jhu.edu/software/stringtie/dl/prepDE.py).^[^ ^78^ ^]^ R package DEseq2^[^ ^79^ ^]^ was used to determine differentially expressed genes and genes with less than 10 counts across all samples were excluded. Gene Ontology (GO) analysis was performed using a custom PERL script. Heat map was created using R package pheatmap.

##### Statistical Analysis

Data for relative expression levels, disease index and calcium influxes were presented as mean ± SD. Two‐tailed Student's *t*‐tests was performed to compare the disease index between the haplotypes of *GhGLR4.8* in the GWAS population. significance was defined as *P* ≤ 0.05. Statistical analysis was carried out using GraphPad Prism software.

##### Data Availability

The raw sequencing data of the 222 accessions genotyped previously are available in the NCBI Sequence Read Archive (SRA) under accession number SRP080913,^[^ ^12^ ^]^ and other 68 accessions are available in the NCBI BioProject under accession number PRJNA579217. The RNA‐seq data for *Fov* cultured in vitro*, Fov*‐inoculated J668 and *Fov7*_KO#5 have been deposited in the NCBI BioProject under accession number PRJNA667289.

## Conflict of Interest

The authors declare no conflict of interest.

## Author Contributions

L.Z. and X.Z. conceived and designed the project. L.Z. designed the experiment. J.M., W.S., X.N., C.Y., J.K., and A.A. prepared the 290 cotton samples and investigated the disease severity in field. S.L. performed data analysis. S.L., X.Z., S.X., T.Q., T.W., Y.W., and Z.Z. performed experiments. J.L. offered resequencing data of a part of cotton accessions. S.L. wrote the manuscript draft. L.Z., S.J.K., K.L., and X.Z. revised it.

## Supporting information

Supporting Information

Supplemental Table 1

Supplemental Table 3

Supplemental Table 7

Supplemental Table 8

Supplemental Table 9

Supplemental Table 10
