# Supplementary material for: A Single‐Nucleotide Mutation in a GLUTAMATE RECEPTOR‐LIKE Gene Confers Resistance to Fusarium Wilt in Gossypium hirsutum
Source: Adv Sci (Weinh). 2021 Feb 19;8(7):2002723. doi: 10.1002/advs.202002723 (PMC8025038; doi:10.1002/advs.202002723)
Supplement: Supplementary file 1 — Supporting Information [file ADVS-8-2002723-s001.pdf]

© 2021 Wiley-VCH GmbH

ADVANCED SCIENCE
Open Access

Supporting Information

for *Adv. Sci.,* DOI: 10.1002/advs.202002723

A single-nucleotide mutation in a GLUTAMATE RECEPTOR-LIKE gene confers resistance to Fusarium wilt in *Gossypium hirsutum*

*Shiming Liu, Xiaojun Zhang, Shenghua Xiao, Jun Ma, Weijun Shi, Tao Qin, Hui Xi, Xinhui Nie, Chunyuan You, Zheng Xu, Tianyi Wang, Yujing Wang, Zhennan Zhang, Jianying Li, Jie Kong, Alifu•Aierxi, Yu Yu, Keith Lindsey, Steven J. Klosterman, Xianlong Zhang and Longfu Zhu**

Supporting Information

A single-nucleotide mutation in a GLUTAMATE RECEPTOR-LIKE gene confers resistance to Fusarium wilt in *Gossypium hirsutum*

Shiming Liu, Xiaojun Zhang, Shenghua Xiao, Jun Ma, Weijun Shi, Tao Qin, Hui Xi, Xinhui Nie, Chunyuan You, Zheng Xu, Tianyi Wang, Yujing Wang, Zhennan Zhang, Jianying Li, Jie Kong, Alifu•Aierxi, Yu Yu, Keith Lindsey, Steven J. Klosterman, Xianlong Zhang and Longfu Zhu[*]

Supplementary Figures and Tables

Supplementary Figure 1

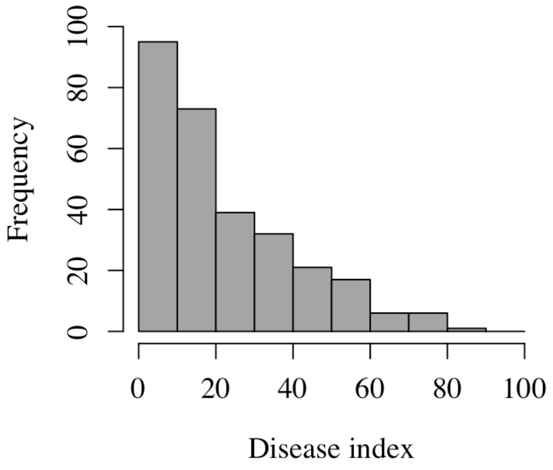

Supplementary Figure 1. Frequency distribution histogram of disease index of the 290 Upland cotton varieties.

Supplementary Figure 2

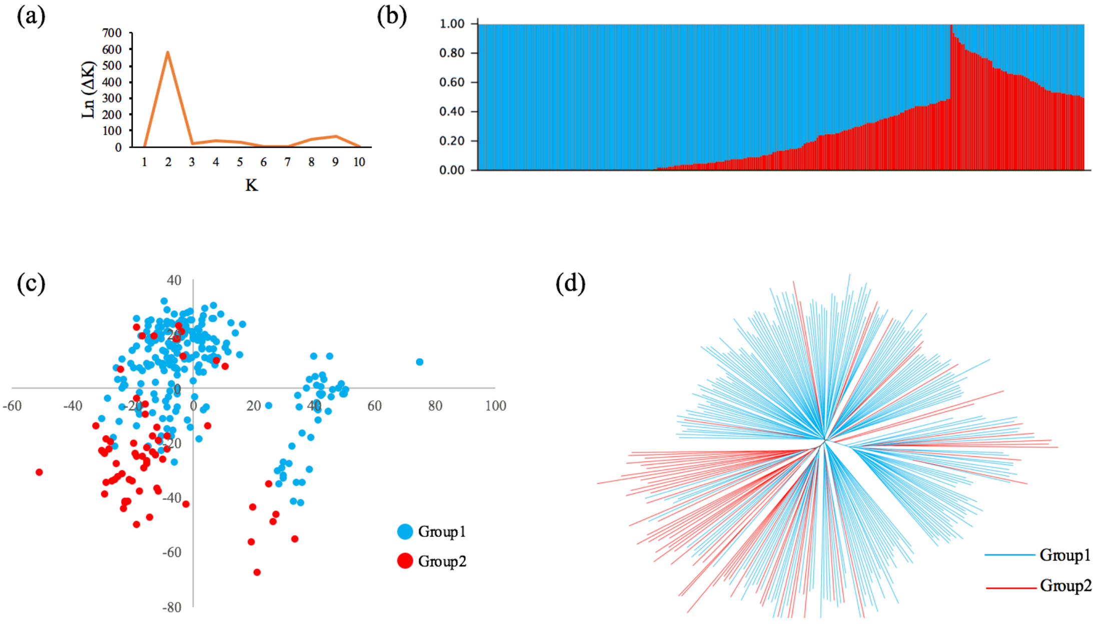

Supplementary Figure 2. Population structure of the 290 cotton accessions. **(a)** Corresponding Ln (ΔK) values when K from 1 to 10. **(b)** When K=2, the population contained 290 cotton accessions was divided into two subpopulations. **(c)** Principal component analysis (PCA) of the population. **(d)** Neighbor-joining tree of the 290 cotton accessions.

Supplementary Figure 3

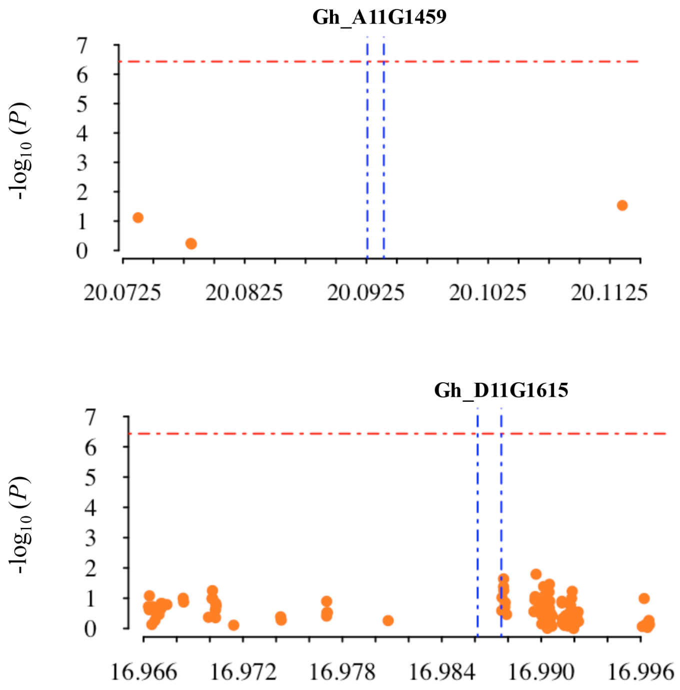

Supplementary Figure 3. The nucleotide variations nearby the *GaGSTF9* homologue in *G. hirsutum* are not associated with Fusarium wilt resistance in Upland cotton. *Gh_A11G1459* and *Gh_D11G1615* are the homologous genes of *GaGSTF9* in A subgenome and D subgenome, respectively. Blue dashed line represents the genome region of the gene. Red dashed line represents the Bonferroni-adjusted significance threshold ($-\log_{10}(P) = 6.43$).

Supplementary Figure 4

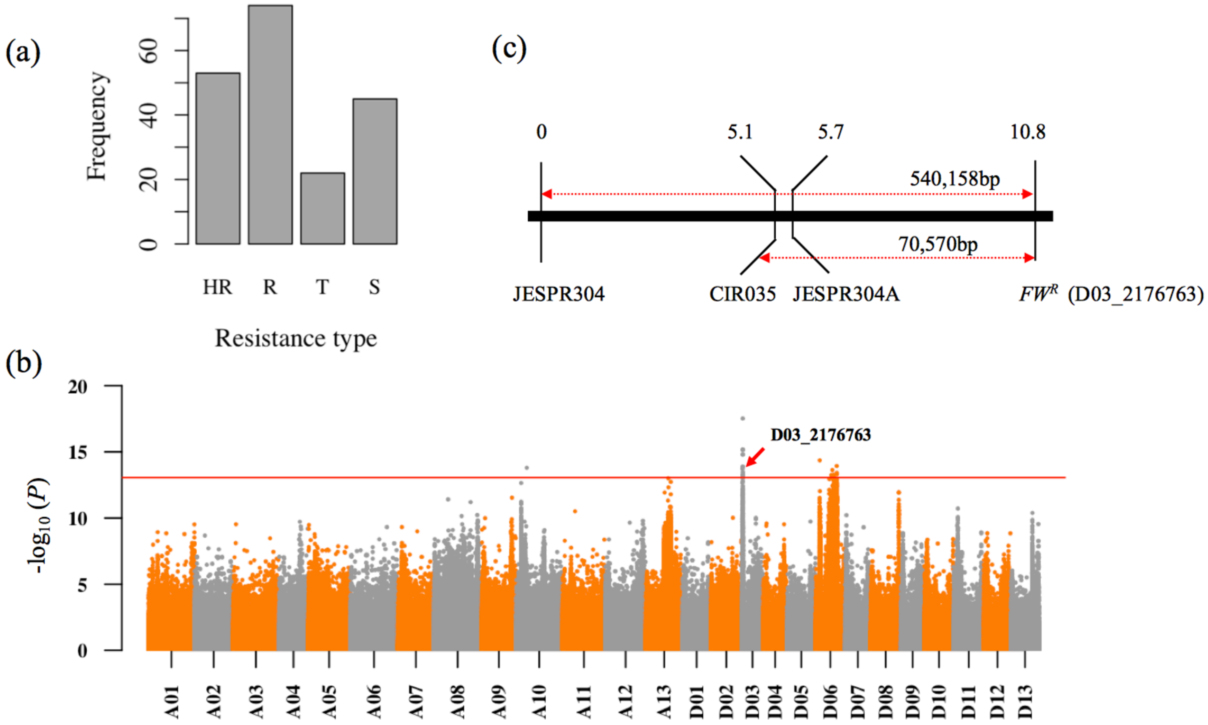

Supplementary Figure 4. GWAS analysis for Fusarium wilt resistance in a population contained 194 Upland cotton cultivars. **(a)** Frequency distribution histogram of disease index of the population. **(b)** Manhattan plot for disease index. The red solid line represents the significance threshold ($-\log_{10}(P) = 13.05$). The significant SNP (D03_2176763) was marked by arrowhead. **(c)** The distance between FW^R-linked SSR markers and D03_2176763 on chromosome D03. Position of FW^R and SSR markers was determined according to Wang.[2] These two SSR markers were mapped to the 'TM-1' reference genome recently assembled by PacBio RSII.[19] The sequence of JESPR304 matches with a genomic region from 1,707,836 bp to 1,707,629 bp, and CIR035 from 2,177,424 bp to 2,177,088 bp on chromosome D03. The SNP D03_2176763 is at the 2,247,994 bp-position of on chromosome D03 in the 'TM-1' genome.

Supplementary Figure 5

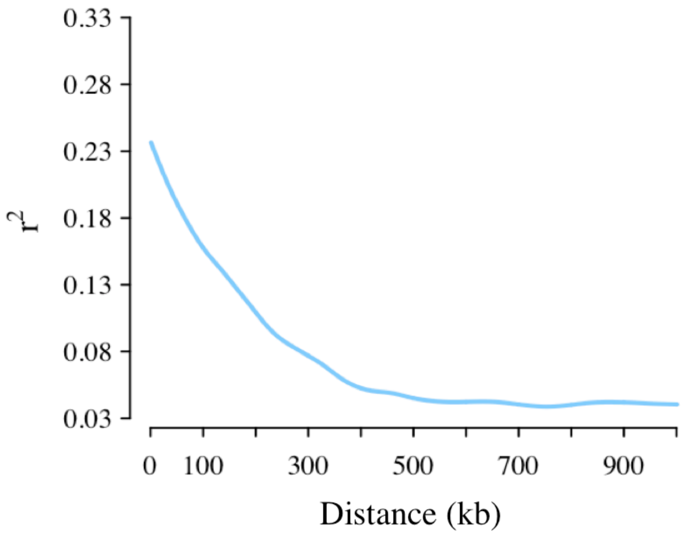

Supplementary Figure 5. Linkage disequilibrium (LD) decay in D03 chromosome. When r^2 is half of the maximum, the corresponding physical distance is set as the extent of LD (200 kb).

Supplementary Figure 6

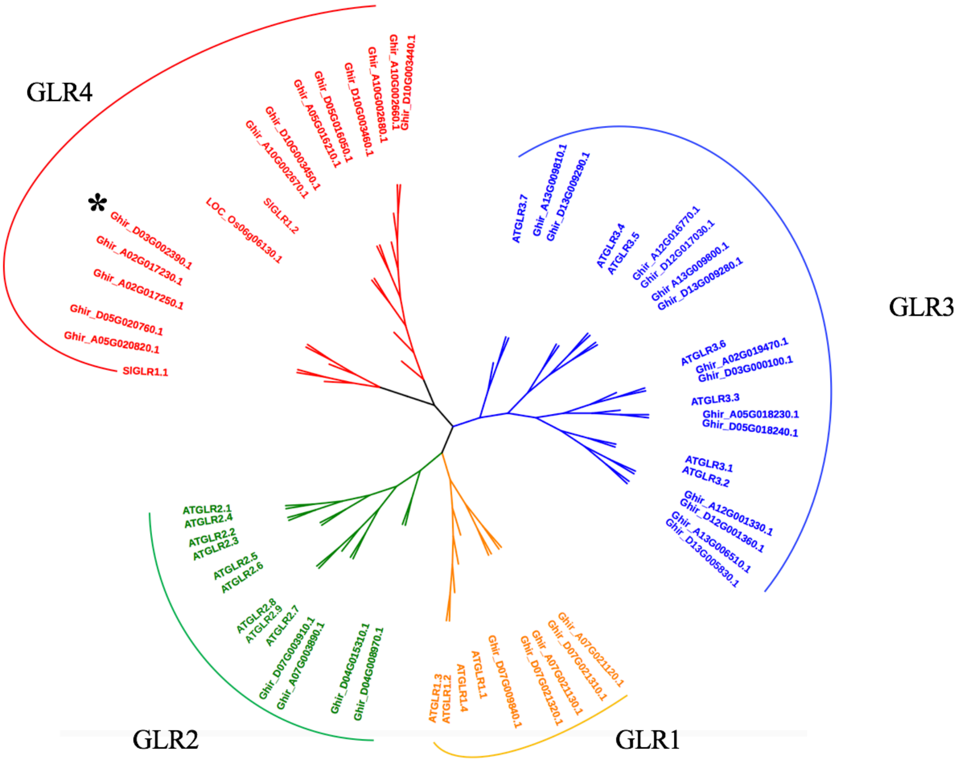

Supplementary Figure 6. Phylogenetic tree of glutamate receptors from *G. hirsutum* (Ghir), *S. lycopersicum* (Sl), *O. sativa* (Os) and *A. thaliana* (AT). Ghir_D03G002390.1 corresponds to Gh_D03G0209 and is marked by asterisk. GLR1、GLR2、GLR3 and GLR4 are indicated by orange, green, purple and red. SlGLR1.1 and SlGLR1.2 are GLRs in *S. lycopersicum*, the accession numbers are AB623193 and AB623194, respectively. LOC_Os06g06130.1 is a GLR in *O. sativa*.

Supplementary Figure 7

```
GhGLR4.8  ............................................................
GluA2     ..................................MQKIMHISVLLSPVLWGLIFG.....
ATGLR1.1  ......................ME.........ILFSISILALLFSGVVAAPS...DDD.
ATGLR1.2  ......................MVRICIQTP..ILLSFLLVLLFFISNCFASSQNNDDD.
ATGLR1.3  ......................MERFCIQTQ..TLLSFLLVLLLFISRSFASTKNDDVDG
ATGLR1.4  ......................MENCMIRNTGYFLTIFFLAFISFAVTCSGTNQKDNVDR
ATGLR2.1  ...........................MKRENNLVLSLLFFVIVFLMQVGEAQN......
ATGLR2.2  ...........................MKNSKLFFR.FLFLFFFFCLESSRGQDN.....
ATGLR2.3  ...........................MRTEKLFF..CILLVFFFCLEFNRGQNN.....
ATGLR2.4  ...........................MKRHLNDVV.LVFLVFIFGVKLGKGQNT.....
ATGLR2.5  ............................................................
ATGLR2.6  ........................MSLFNHLLSRALPLWLLFFINFLVLLGKSQQ.....
ATGLR2.7  .....................MKVMNPRKTNNTFMYYFVLFVCGFVLMEGCLGQN.....
ATGLR2.8  ........................MNPKKNNNTFLSY...FVCLFLLLEVGLGQN.....
ATGLR2.9  ...........................MKTNNTFLSY...FVCGFLLMGVGLGQN.....
ATGLR3.1  ...........................MLSSMNWVLLSFIIVLGGGLLLSEGASS.....
ATGLR3.2  ...........................MFWVL..VLLSFIVLIGDG.MISEGAG......
ATGLR3.3  ...............................MKQLWTFFFLS.FLCSGLFRRTHS.....
ATGLR3.4  MGFLVMIREVSMAKAIRVVLLCVSVLWVVPKECACRSNFSRNSSSSSSSSLRPLR.....
ATGLR3.5  .................................................MILSLE.....
ATGLR3.6  ...............................MKWFLLMLIICNAVPLQGLTKIVS.....
ATGLR3.7  ..............................MGLGIDPSVAITALIVVILVVPMDC.....
```

```
GhGLR4.8  ............................................................
GluA2     ..VSSNSIQIGGLFPRG...ADQEYSAFRVGMVQFSTSEFRLTPHIDNLEVANS.....F
ATGLR1.1  ...VFEEVRVGLVVDLSSIQGKILETSFNLALSDFYGINNGYR.TRVSVLVRDSQGDPII
ATGLR1.2  ...KRIRVRVGLVLDLGSVEGKIVRSSVSMALSDFYDNHNDYK.TRLSLLVRDSHGEPLL
ATGLR1.3  G..KRVQIRVGLVLDLGSLKGKIVKNSVSMALSYFYAIHNDYK.TRVSVSLRNSHGEPLL
ATGLR1.4  LPVVYEDVRIGLVVDMGSMEGKLVTTSISMALSDFYHVNNGYR.TRVSVLSRDSHGDPLQ
ATGLR2.1  ...RITNVNVGIVNDIGTAYSNMTLLCINMSLSDFYSSHPETQ.TRLVTTVVDSKNDVVT
ATGLR2.2  ...GKTQVNIGVVSDVGTSYPDVAMLCINMSLADFYSSRPQFQ.TRLVVNVGDSKNDVVG
ATGLR2.3  ...GKTLVDVGVVTDVDTSHSKVVMLCINMSISDFYSSNPQFE.TRLVVNVGDSKSDVVG
ATGLR2.4  ...TIQVINVGVVTDVGTTASNLSLLAINMSLSDFYSSRPESR.TRLLLNFADSRDDVVG
ATGLR2.5  .............................MSLSEFYNTHNGFK.TRIVLNVRDSKQTVVG
ATGLR2.6  ...EVLQVQVGIVLDTNATLAALSLRAINMSLSEFYNTHNGFK.TRIVLNIRDSKRTVVG
ATGLR2.7  ...QTTEIKVGVVLDLHTSFSKLCLTSINISLSDFYKYHSDYT.TRLAIHIRDSMEDVVQ
ATGLR2.8  ...QISEIKVGVVLDLNTTFSKICLTSINLALSDFYKDHPNYR.TRLALHVRDSMKDTVQ
ATGLR2.9  ...QTSEIKVGVVLDLNTTFSKICLTSIKMAVSDFYADHPNYL.TRLTLHVRDSMEDTVQ
ATGLR3.1  ..SRPPVIKVGAIFGLNTMYGETANIAFKAAEEDVNSDPSFLGGSKLRILMNDAKRSGFL
ATGLR3.2  ..LRPRYVDVGAIFSLGTLQGEVTNIAMKAAEEDVNSDPSFLGGSKLRITTYDAKRNGFL
ATGLR3.3  ..EKPKVVKIGSIFSFDSVIGKVAKIAIDEAVKDVNSNPDILSGTKFSVSMQNSNCSGFM
ATGLR3.4  ..QRPSSVNVGALFTYDSFIGRAAKPAVKAAMDDVNADQSVLKGIKLNIIFQDSNCSGFI
ATGLR3.5  ..ERPN..............LRLWRPLKTLMLTRVSSGAPSLILSSRTLIAVD.......
ATGLR3.6  ..ARPQVVNIGSVFTFNSLIGKVIKVAMDAAVEDVNASPSILNTTTLRIIMHDTKYNGFM
ATGLR3.7  ..QRPQLVNIGAVFAFDSVIGRAAKVALEAAVSDVNNDKSFLKETELRLLMEDSACNVFR
```

```
GhGLR4.8  ............................................................
GluA2     AVTNAFCSQFSRGVYAIFGFYDKKSVNTITSFCGTLHVSFIT....PSFPTDGTHPFVIQ
ATGLR1.1  ALAAATDLLKNAKAEAIVGAQSLQEAKLLATISEKAKVPVIS.TFLPNTLSLKKYDNFIQ
ATGLR1.2  ALDSVVDLLQTEGVQAIIGGNSLLEAKLLAELGEKARVPVIS.LNSPMSLSLSKYTHLIQ
ATGLR1.3  ALASAVDLLKTEGVEAIIGGNSLLEAKLLGELGEKARVPMIS.LDSPFSLSLSKYTHLIQ
ATGLR1.4  ALAAAMDLLQTEQVEALVGGQSLLEAKNLAELGEKTKVPVISSFQVPSSLSLAKYNYFIQ
ATGLR2.1  AAAAALDLITNKEVKAILGPWTSMQAQFMIEMGQKSQVPIVTYSATSPSLASIRSQYFFR
ATGLR2.2  AATAAIDLIKNKQVKAILGPWTSMQAHFLIEIGQKSRVPVVSYSATSPSLTSLRSPYFFR
ATGLR2.3  AAIAALDLIKNKQVKAILGPWTSMQAHFLIEIGQKSRVPIVSYSATSPILTSLRSPYFLR
ATGLR2.4  AAAAALDLIKNKEVKAILGPRTTMQASFVIEVGQKSQVPIISFSATSPFLDSGRSPYFFR
ATGLR2.5  AAASALYLIKKREVVAIIGPGTSMQAPFLINLGNQSKVPIISFSATSPLLDSLRSPYFIR
ATGLR2.6  AAASALYLIKKREVVAIIGPGNSMQAPFLINLGNQSQVPIISFSASSPVLDSLRSPYFIR
ATGLR2.7  ASSAALDLIKNEQVSAIIGPRTSMQAEFMIRLADKSQVPTITFSATCPLLTSINSPYFVR
ATGLR2.8  ASAAALDLIQNEQVSAIIGPIDSMQAKFMIKLANKTQVPTISFSATSPLLTSIKSDYFVR
ATGLR2.9  ASAAALDLIKTEQVSAIIGPINSMQADFMIKLANKTQVPTITFSATSPLLTSIKSPYFVR
ATGLR3.1  SIMGALQFMET.DVVAIIGPQTSIMAHVLSHLANELTVPMLSFTALDPTLSPLQFPFFVQ
ATGLR3.2  TIMGALQFMET.DAVAIIGPQTSIMAHVLSHLANELSVPMLSFTALDPSLSALQFPFFVQ
ATGLR3.3  GMVEALRFMEK.DIVGIIGPQCSVVAHMISHMANELRVPLLSFAVTDPVMSPLQFPYFIR
ATGLR3.4  GTMGALQLMEN.KVVAAIGPQSSGIAHMISYVANELHVPLLSFGATDPTLSSLQFPYFLR
ATGLR3.5  .LLAPWELMEN.KVVAAIGPQSSGIGHIISHVANELHVPFLSFAATDPTLSSLQYPYFLR
ATGLR3.6  SIMEPLQFMES.ETVAIIGPQRSTTARVVAHVATELKIPILSFSATDPTMSPLQFPFFIR
ATGLR3.7  GSFGAFELLEK.EVVAMIGPISSSVAHTISDIAKGLHFPLVSFAATDPTLSALQFPFFLR
```

```
GhGLR4.8   ............................................................
GluA2      MRPDLKG..ALLSLLIEYYQWDKFAYLY.DSDRGLSTLQAVLDSAAEKKWQVTAINVGNI
ATGLR1.1   WTHDTTSEAKGITSLIQDFSCKSVVVIYEDADDWSESLQILVENFQDKGIYIARSASFAV
ATGLR1.2   ATHNSASEVKGITAFLHGFDWNSVALVLEDHDDWRESMHFMVDHFHENNVHVQSKVAFSV
ATGLR1.3   ATHDSTSEAKGITSFINVFDWNSVALVYEDHDDWRESMQLLVEHFHENGVRVQSKVGFTV
ATGLR1.4   ATHDTSSEAKGIAALFSNFDWRTAVLIYEDDDDWRESIQPLVGHFQQNAIHIEYKAEFSV
ATGLR2.1   ATYDDSSQVHAIKEIIKLFGWREVAPVYVDDTFGEGIMPRLTDVLQEINVRIPYRTVISP
ATGLR2.2   ATYEDSSQVHAIKAIIKLFGWREVVPVYIDNTFGEGIMPRLTDSLQDINVRIPYRSVIPL
ATGLR2.3   ATYEDSFQVQPIKAIIKLFGWREVVPVYIDNTFGEGIMPRLTDALQDINVRIPYRSVIAI
ATGLR2.4   STYDDSSQVQAISEIIKVFGWREVVPVYENNAFGEGIMPGLTDALQAINIRIPYRTVISP
ATGLR2.5   ATHDDSSQVQAISAIIESFRWREVVPIYVDNEFGEGILPNLVDAFQEINVRIRYRSAISL
ATGLR2.6   ATHDDSSQVHAISAIIESFRWREVVPIYADNEFGEGILPYLVDAFQEINVRIRYRSAISV
ATGLR2.7   ATLDDSSQVKAIAAIVKSFGWRNVVAIYVDNEFGEGILPLLTDALQDVQAFVVNRCLIPQ
ATGLR2.8   GTIDDSYQVKAIAAIFESFGWRSVVAIYVDNELGEGIMPYLFDALQDVQ...VDRSVIPS
ATGLR2.9   ATIDDSSQVRAIASIFKFFRWRRVVAIYVDNEFGEGFMPFLFDALQDVE...VKRSVIPP
ATGLR3.1   TAPSDLFLMRAIAEMITYYGWSDVVALYNDDDNSRNGVTALGDELEERRCKISYKAVLPL
ATGLR3.2   TAPSDLFLMRAIAEMISYYGWSEVIALYNDDDNSRNGITALGDELEGRRCKISYKAVLPL
ATGLR3.3   TTQSDLYQMDAIASIVDFYGWKEVIAVFVDDDFGRNGVAALNDKLASRRLRITYKAGLHP
ATGLR3.4   TTQNDYFQMHAIADFLSYSGWRQVIAIFVDDECGRNGISVLGDVLAKKRSRISYKAAITP
ATGLR3.5   TTQNDYFQMNAITDFVSYFRWREVVAIFVDDEYGRNGISVLGDALAKKRAKISYKAAFPP
ATGLR3.6   TSQNDLFQMAAIADIVQFYRWREVVAIYGDDDYGRNGVAALGDRLSEKRCRISYKAALPP
ATGLR3.7   TTPNDAHQMSALVDLINFYGWKEVISVYSDDELGRNGVSALDDELYKKRSRISYKVPLSV
```

```
           1        10        20        30        40        50
GhGLR4.8   .MSAKIFAFVLLLILSSKCSGIRDGDDQKDTEDDSCLMSCNTASDAHHIQLKFNHSPGNV
GluA2      N.NDKKDEMYRSLFQDLELKKERRVILDCERDKVNDIVDQVITIGKHVKGYHYIIANLGF
ATGLR1.1   ..SSSGENHMMNQLRKLKVSRASVFVVHMSEILVSRLFQCVEKLGLMEEAFAWILTARTM
ATGLR1.2   ..TSS.EDSLMDRLRELKDLGTTVFVVHLSEVIATRLFPCAEKLGMMGEGFAWILTSRSM
ATGLR1.3   ..SSS.EDFVMGRLQQLKDLGTTVFVVHLSEVIATHLFPCARRLGLMGDGFVWILTAKTM
ATGLR1.4   ..SSN.EECIMKQLRKFKASGIRIFVAHISERIANRLFPCARRLGMMEEGYAWILTARSM
ATGLR2.1   ..NAT.DDEISVELLRMMTLPTRVFVVHLVELLASRFFAKATEIGLMKQGYVWILTNTIT
ATGLR2.2   ..NAT.DQDISVELLKMMNMPTRVFIVHMSSSLASTVFIKAKELGLMKPGYVWILTNGVM
ATGLR2.3   ..NAT.DHEISVELLKMMNMPTRVFLVHMYYDLASRFFIKAKELGLMEPGYVWILTNGVI
ATGLR2.4   ..NAT.DDEISVDLLKLMTKPTRVFVVHMNRFLASRVFSKARETGLMKQGYAWILTNGVI
ATGLR2.5   ..HYS.DDQIKKELYKLMTMPTRVFIVHMLPDLGSRLFSIAKEIDMLSKGYVWIVTNGIA
ATGLR2.6   ..HST.DDLVKKELYKLMTMPTRVFIVHMLPDLGSRLFSIAKEIGMMTKGYVWIVTNGIA
ATGLR2.7   ..EAN.DDQILKELYKLMTMQTRVFVVHMPPTLGFRFFQKAREIGMMEEGYVWLLTDGVM
ATGLR2.8   ..EAN.DDQILKELYKLMTRQTRVFVVHMASRLASRIFEKATEIGMMEEGYVWLMTNGMT
ATGLR2.9   ..EAI.DDEIQKELRKLMERQARVFVVHMESSLALRVFQIARDIGMMEEGYVWLMTNGMT
ATGLR3.1   DVVITSPVEIIEELIKIRGMESRVIVVNTFPNTGKMIFKEAERLGMMEKGYVWIATTWLS
ATGLR3.2   DVVITSPREIINELVKIQGMESRVIIVNTFPKTGKKIFEEAQKLGMMEKGYVWIATTWLT
ATGLR3.3   DTAVNKN.EIMNMLIKIMLLQPRIVVIHVYSELGFAVFKEAKYLGMMGNGYVWIATDWLS
ATGLR3.4   GADSSS...IRDLLVSVNLMESRVFVVHVNPDSGLNVFSVAKSLGMMASGYVWIATDWLP
ATGLR3.5   GADNSS...ISDLLASVNLMESRIFVVHVNPDSGLNIFSVAKSLGMMGSGYVWITTDWLL
ATGLR3.6   ..APTRE.NITDLLIKVALSESRIIVVHASFIWGLELFNVARNLGMMSTGYVWIATNWLS
ATGLR3.7   HSDEKF...LTNALNKSKSIGPRVYILHFGPDPLLRIFDIAQKLQMMTHEYVWLATDWLS
```

SNP1

```
          60      ↓  70        80        90             100
GhGLR4.8   TPIDLY.........DDYQNYVAIKNLTSHRLPILCTVTSYETAFS.........AEINAF
GluA2      TDGDLLK.....IQFGGANVSGFQIVDYDDSLVSKFIERWS.TLEEKEYPGAHTTTIKYT
ATGLR1.1   NYLEHF.....AITRSMQGVIGFKSYIPVSEEVKNFTSRLRKRMGDDTET...EHSSVII
ATGLR1.2   SSFHDQFI.DDLTKEAMEGVVGFKSYIPMSKELHNFTLRWRKTLPVEEVTGSEITRLSIS
ATGLR1.3   NSFHEN.I.DDFTKQAMEGVVGFKSYIPMSIELQNFTLRWRKSLPVEEA...ELTRLSIS
ATGLR1.4   NNFQDT...NYLAKEEMEGVIGFKSYIPLTEELHNFTLRWKRSLRLEEV....VTRMSVC
ATGLR2.1   DVLSIMN...ETEIETMQGVLGVKTYVPRSKELENFRSRWTKRFP........ISDLNVY
ATGLR2.2   DGLRSIN...ETGIEAMEGVLGIKTYIPKSKDLETFRSRWKRRFP........QMELNVY
ATGLR2.3   DDLSLIN...ETAVEAMEGVLGIKTYIPKSPDLEKFRSRWRSLFP........RVELSVY
ATGLR2.4   DHLVLMN...GTDIEAMQGVIGIRTHFPISEELQTFRSRLAKAFP........VSELNIY
ATGLR2.5   DLMSIMG...ESSLVNMHGVLGVKTYFAKSKELLHLEARWQKRFG........GEELNNF
ATGLR2.6   DQMSVMG...ESSLENMHGVLGVKTYFSRSKELMYLETRWRKRFG........GEELNNF
ATGLR2.7   NLLKSNER..GSSLENMQGVLGVRSHIPKSKKLKNFRLRWEKMFPKKGN....DEEMNIF
ATGLR2.8   HMMRHIHH..GRSLNTIDGVLGVRSHVPKSKGLEDFRLRWKRNFKKENPW..LRDDLSIF
ATGLR2.9   HMMRHINN..GRSLNTIEGVLGVRSHVPKSKELGDFRLRWKRTFEKENPS..MRDDLNVF
ATGLR3.1   SVLDSNLPLDTKLVN...GVLTLRLHTPDSRKKRDFAARWKNKLSNNK.....TIGLNVY
ATGLR3.2   SLLDSVNPLPAKTAESLRGVLTLRIHTPNSKKKKDFVARWN.KLSNG......TVGLNVY
ATGLR3.3   TNLDSSSPLPAERLETIQGVLVLRPHTPDSDFKREFFKRWR.KMSGA......SLALNTY
ATGLR3.4   TAMDSMEHVDSDTMDLLQGVVAFRHYTIESSVKRQFMARWK.NLR.......PNDGFNSY
ATGLR3.5   TALDSMEPLDPRALDLLQGVVAFRHYTPESDNKRQFKGRWK.NLRFKESLK.SDDGFNSY
ATGLR3.6   TIIDTDSPLPLDTINNIQGVITLRLHTPNSIMKQNFVQRWH.NLT........HVGLSTY
ATGLR3.7   VTLDSLS..DKGTLKRLEGVVGLRQHIPESVKMEHFTHKLQSNRS...........MNAY
```

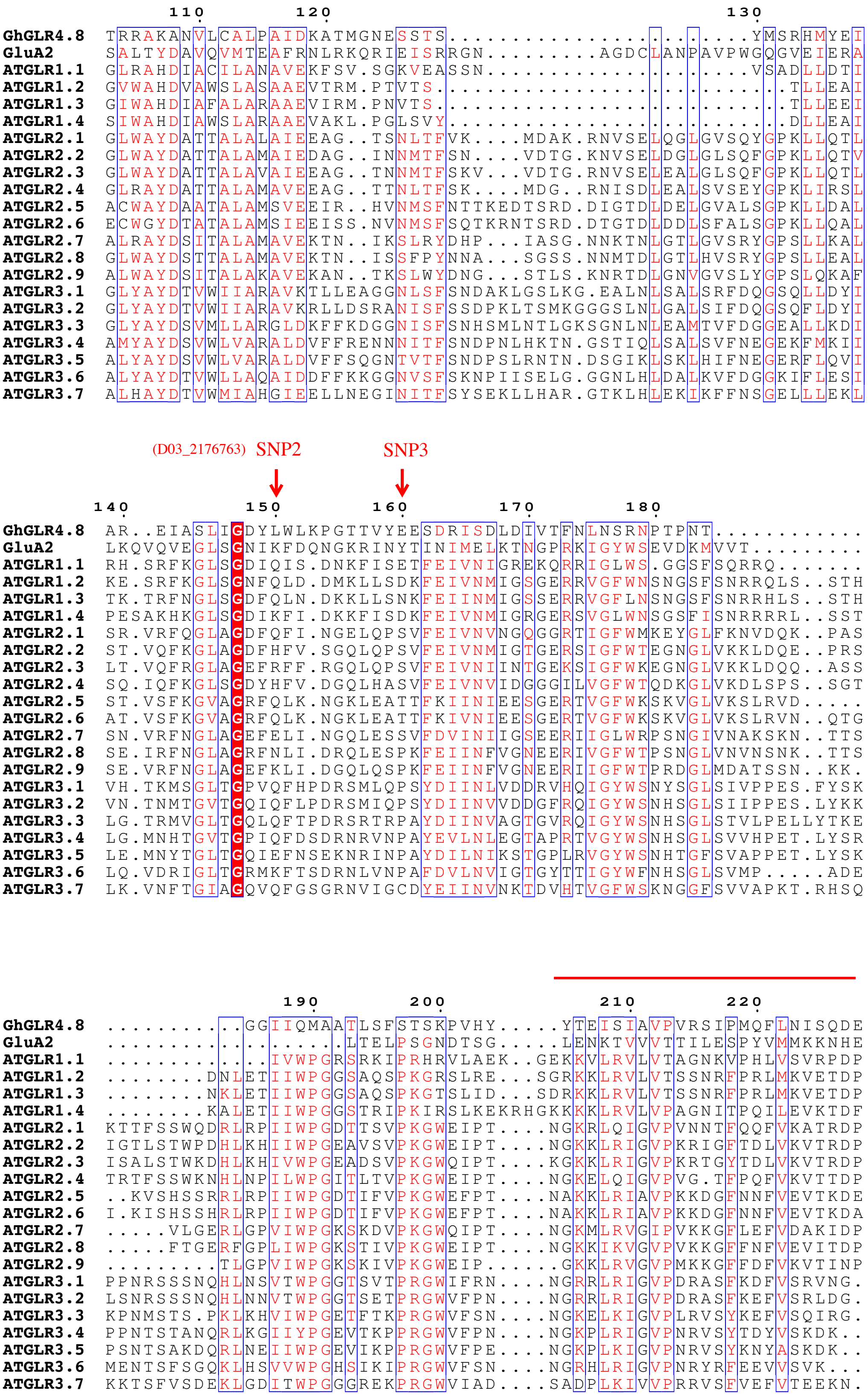
110 120 130
GhGLR4.8 TRRAKANVLCALPAIDKATMGNESSTS........................YMSRHMYEI
GluA2 SALTYDAVQVMTEAFRNLRKQRIEISRRGN.........AGDCLANPAVPWGQGVEIERA
ATGLR1.1 GLRAHDIACILANAVEKFSVSGKVEASSN.....................VSADLLDTI
ATGLR1.2 GVWAHDVAWSLASAAEVTRM.PTVTS.........................TLLEAI
ATGLR1.3 GIWAHDIAFALARAAEVIRM.PNVTS.........................TLLEEI
ATGLR1.4 SIWAHDIAWSLARAAEVAKL.PGLSVY........................DLLEAI
ATGLR2.1 GLWAYDATTALALAIEEAG..TSNLTFVK....MDAK.RNVSELQGLGVSQYGPKLLQTL
ATGLR2.2 GLWAYDATTALAMAIEDAG..INNMTFSN....VDTG.KNVSELDGLGLSQFGPKLLQTV
ATGLR2.3 GLWAYDATTALAVAIEEAG..TNNMTFSKV...VDTG.RNVSELEALGLSQFGPKLLQTL
ATGLR2.4 GLRAYDATTALAMAVEEAG..TTNLTFSK....MDG..RNISDLEALSVSEYGPKLIRSL
ATGLR2.5 ACWAYDAATALAMSVEEIR..HVNMSFNTTKEDTSRD.DIGTDLDELGVALSGPKLLDAL
ATGLR2.6 ECWGYDTATALAMSIEEISS.NVNMSFSQTKRNTSRD.DTGTDLDDLSFALSGPKLLQAL
ATGLR2.7 ALRAYDSITALAMAVEKTN..IKSLRYDHP...IASG.NNKTNLGTLGVSRYGPSLLKAL
ATGLR2.8 GLWAYDSTTALAMAVEKTN..ISSFPYNNA...SGSS.NNMTDLGTLHVSRYGPSLLEAL
ATGLR2.9 ALWAYDSITALAKAVEKAN..TKSLWYDNG...STLS.KNRTDLGNVGVSLYGPSLQKAF
ATGLR3.1 GLYAYDTVWIIARAVKTLLEAGGNLSFSNDAKLGSLKG.EALNLSALSRFDQGSQLLDYI
ATGLR3.2 GLYAYDTVWIIARAVKRLLDSRANISFSSDPKLTSMKGGGSLNLGALSIFDQGSQFLDYI
ATGLR3.3 GLYAYDSVMLLARGLDKFFKDGGNISFSNHSMLNTLGKSGNLNLEAMTVFDGGEALLKDI
ATGLR3.4 AMYAYDSVWLVARALDVFFRENNNITFSNDPNLHKTN.GSTIQLSALSVFNEGEKFMKII
ATGLR3.5 ALYAYDSVWLVARALDVFFSQGNTVTFSNDPSLRNTN.DSGIKLSKLHIFNEGERFLQVI
ATGLR3.6 ALYAYDTVWLLAQAIDDFFKKGGNVSFSKNPIISELG.GGNLHLDALKVFDGGKIFLESI
ATGLR3.7 ALHAYDTVWMIAHGIEELLNEGINITFSYSEKLLHAR.GTKLHLEKIKFFNSGELLLEKL
(D03_2176763) SNP2
SNP3
140 150 160 170 180
GhGLR4.8 AR..EIASLIGDYLWLKPGTTVYEESDRISDLDIVTFNLNSRNPTPNT............
GluA2 LKQVQVEGLSGNIKFDQNGKRINYTINIMELKTNGPRKIGYWSEVDKMVVT.........
ATGLR1.1 RH.SRFKGLSGDIQIS.DNKFISETFEIVNIGREKQRRIGLWS.GGSFSQRRQ.......
ATGLR1.2 KE.SRFKGLSGNFQLD.DMKLLSDKFEIVNMIGSGERRVGFWNSNGSFSNRRQLS..STH
ATGLR1.3 TK.TRFNGLSGDFQLN.DKKLLSNKFEIINMIGSSERRVGFLNSNGSFSNRRHLS..STH
ATGLR1.4 PESAKHKGLSGDIKFI.DKKFISDKFEIVNMIGRGERSVGLWNSGSFISNRRRRL..SST
ATGLR2.1 SR.VRFQGLAGDFQFI.NGELQPSVFEIVNVNGQGGRTIGFWMKEYGLFKNVDQK..PAS
ATGLR2.2 ST.VQFKGLAGDFHFV.SGQLQPSVFEIVNMIGTGERSIGFWTEGNGLVKKLDQE..PRS
ATGLR2.3 LT.VQFRGLAGEFRFF.RGQLQPSVFEIVNIINTGEKSIGFWKEGNGLVKKLDQQ..ASS
ATGLR2.4 SQ.IQFKGLSGDYHFV.DGQLHASVFEIVNVIDGGGILVGFWTQDKGLVKDLSPS..SGT
ATGLR2.5 ST.VSFKGVAGRFQLK.NGKLEATTFKIINIEESGERTVGFWKSKVGLVKSLRVD.....
ATGLR2.6 AT.VSFKGVAGRFQLK.NGKLEATTFKIVNIEESGERTVGFWKSKVGLVKSLRVN..QTG
ATGLR2.7 SN.VRFNGLAGEFELI.NGQLESSVFDVINIIGSEERIIGLWRPSNGIVNAKSKN..TTS
ATGLR2.8 SE.IRFNGLAGRFNLI.DRQLESPKFEIINFVGNEERIVGFWTPSNGLVNVNSNK..TTS
ATGLR2.9 SE.VRFNGLAGEFKLI.DGQLQSPKFEIINFVGNEERIIGFWTPRDGLMDATSSN..KK.
ATGLR3.1 VH.TKMSGLTGPVQFHPDRSMLQPSYDIINLVDDRVHQIGYWSNYSGLSIVPPES.FYSK
ATGLR3.2 VN.TNMTGVTGQIQFLPDRSMIQPSYDIINVVDDGFRQIGYWSNHSGLSIIPPES.LYKK
ATGLR3.3 LG.TRMVGLTGQLQFTPDRSRTRPAYDIINVAGTGVRQIGYWSNHSGLSTVLPELLYTKE
ATGLR3.4 LG.MNHTGVTGPIQFDSDRNRVNPAYEVLNLEGTAPRTVGYWSNHSGLSVVHPET.LYSR
ATGLR3.5 LE.MNYTGLTGQIEFNSEKNRINPAYDILNIKSTGPLRVGYWSNHTGFSVAPPET.LYSK
ATGLR3.6 LQ.VDRIGLTGRMKFTSDRNLVNPAFDVLNVIGTGYTTIGYWFNHSGLSVMP.....ADE
ATGLR3.7 LK.VNFTGIAGQVQFGSGRNVIGCDYEIINVNKTDVHTVGFWSKNGGFSVVAPKT.RHSQ
190 200 210 220
GhGLR4.8 ...........GGIIQMAATLSFSTSKPVHY.....YTEISIAVPVRSIPMQFLNISQDE
GluA2 .................LTELPSGNDTSG.....LENKTVVVTTILESPYVMMKKNHE
ATGLR1.1 ...............IVWPGRSRKIPRHRVLAEK...GEKKVLRVLVTAGNKVPHLVSVRPDP
ATGLR1.2 ........DNLETIIWPGGSAQSPKGRSLRE...SGRKKLRVLVTSSNRFPRLMKVETDP
ATGLR1.3 ........NKLETIIWPGGSAQSPKGTSLID...SDRKKLRVLVTSSNRFPRLMKVETDP
ATGLR1.4 ........KALETIIWPGGSTRIPKIRSLKEKRHGKKKKLRVLVPAGNITPQILEVKTDF
ATGLR2.1 KTTFSSWQDRLRPIIWPGDTTSVPKGWEIPT....NGKRLQIGVPVNNTFQQFVKATRDP
ATGLR2.2 IGTLSTWPDHLKHIIWPGEAVSVPKGWEIPT....NGKKLRIGVPKRIGFTDLVKVTRDP
ATGLR2.3 ISALSTWKDHLKHIVWPGEADSVPKGWQIPT....KGKKLRIGVPKRTGYTDLVKVTRDP
ATGLR2.4 TRTFSSWKNHLNPILWPGITLTVPKGWEIPT....NGKELQIGVPVG.TFPQFVKVTTDP
ATGLR2.5 ..KVSHSSRRLRPIIWPGDTIFVPKGWEFPT....NAKKLRIAVPKKDGFNNFVEVTKDE
ATGLR2.6 I.KISHSSHRLRPIIWPGDTIFVPKGWEFPT....NAKKLRIAVPKKDGFNNFVEVTKDA
ATGLR2.7 .....VLGERLGPVIWPGKSKDVPKGWQIPT....NGKMLRVGIPVKKGFLEFVDAKIDP
ATGLR2.8 .....FTGERFGPLIWPGKSTIVPKGWEIPT....NGKKIKVGVPVKKGFFNFVEVITDP
ATGLR2.9 .........TLGPVIWPGKSKIVPKGWEIP.....GKKLRVGVPMKKGFFDFVKVTINP
ATGLR3.1 PPNRSSSNQHLNSVTWPGGTSVTPRGWIFRN....NGRRLRIGVPDRASFKDFVSRVNG.
ATGLR3.2 LSNRSSSNQHLNNVTWPGGTSETPRGWVFPN....NGRRLRIGVPDRASFKEFVSRLDG.
ATGLR3.3 KPNMSTS.PKLKHVIWPGETFTKPRGWVFSN....NGKELKIGVPLRVSYKEFVSQIRG.
ATGLR3.4 PPNTSTANQRLKGIIYPGEVTKPPRGWVFPN....NGKPLKIGVPNRVSYTDYVSKDK..
ATGLR3.5 PSNTSAKDQRLNEIIWPGEVIKPPRGWVFPE....NGKPLKIGVPNRVSYKNYASKDK..
ATGLR3.6 MENTSFSGQKLHSVVWPGHSIKIPRGWVFSN....NGRHLRIGVPNRYRFEEVVSVK...
ATGLR3.7 KKTSFVSDEKLGDITWPGGGREKPRGWVIAD....SADPLKIVVPRRVSFVEFVTEEKN.

S1

```
           230        240        250        260                 270
GhGLR4.8   KNHNEAQITGFWTDLFKEAVAVMPINTTYKLVPFY..GSD.......DQLFKALVRR...
GluA2      MLEGNERYEGYCVDLAAEIAKHCGFKYKLTIVGDGKYGARDADTKIWNGMVGELVYG...
ATGLR1.1   ETGVN.TVSGFCVEVFKTCIAPFNYELEFIPY...........RGNNDNLAYLLSTQR.D
ATGLR1.2   ITHEITIVEGFCIEVFQASIAPFNYEVEYIRW.....LN....GTNYTKLAYALHSQK.D
ATGLR1.3   VTNEL.IVEGFCIEVFRASISPFNYEVEYIPW.....LN....GSNYDNLAYALHSQK.D
ATGLR1.4   KTGVT.AATGYCIDVFETSILPFNYEVEYIPWPGA..IN....YKNYNDLVYTLYSQK.D
ATGLR2.1   ITNST.IFSGFSIDYFEAVIQAIPYDISYDFIPFQ..DGG......YDALVYQVYLG...
ATGLR2.2   ITNST.VVKGFCIDFFEAVIQAMPYDVSYEFFPFE..KPNGEPAGNHNDLVHQVYLG...
ATGLR2.3   ITNST.VVTGFCIDFFEAVIRELPYDVSYEFIPFE..KPDGKTAGNYNDLVYQVYLG...
ATGLR2.4   LTHET.IVTGFCIDFFEAVIQAMPYDVSHRFIPFG..DD...........DGKTN...
ATGLR2.5   NTNVP.TVTGFCIDVFNTVMSQMPYAVSYEYIPFD..TPDGKPRGSYDEMVYNVFLG...
ATGLR2.6   NTNAP.TITGFCIDVFDTAMRQMPYAVPYEYIPFE..TPDGKPRGSYDEMVYHVFLG...
ATGLR2.7   ISNAM.TPTGYCIEIFEAVLKKLPYSVIPKYIAFL..SPDEN....YDEMVYQVYTG...
ATGLR2.8   ITNIT.TPKGYAIDIFEAALKKLPYSVIPQYYRFE..SPDDD.....YDDLVYKVDNG...
ATGLR2.9   ITNKK.TPTGYAIEIFEAALKELPYLVIPEYVSFE..SPNN.....YNNLVYQVYDK...
ATGLR3.1   ...SSNKVQGYCIDVFEAAVKLLSYPVPHEFIFFG..DGLTN..PNYNELVNKVTTGVD.
ATGLR3.2   ...S.NKVQGYAIDVFEAAVKLISYPVPHEFVLFG..DGLKN..PNFNEFVNNVTIGV..
ATGLR3.3   ...TENMFKGFCIDVFTAAVNLLPYAVPVKFIPYG..NGKEN..PSYTHMVEMITTGN..
ATGLR3.4   ...NPPGVRGYCIDVFEAAIELLPYPVPRTYILYG..DGKRN..PSYDNLVNEVVADN..
ATGLR3.5   ...NPLGVKGFCIDIFEAAIQLLPYPVPRTYILYG..DGKKN..PSYDNLISEVAANI..
ATGLR3.6   ...SNGMITGFCVDVFIAAINLLPYAVPFELVAFG..NGHDN..PSNSELVRLITTGV..
ATGLR3.7   ...SSHRIQGFCIDVFIEALKFVPYSVPYIFEPFG..NGHSS..PNYNHLIQMVTDGV..
```

```
             280        290        300        310         320        330
GhGLR4.8   TFDAAIGLTVMTRKGSELLEFSYPYFEVGPMLVMKEKPEPN.QVFSFMMPFTNEMWCTLA
GluA2      KADIAIAPLTITLVREEVIDFSKPFMSLGISIMIKKPQKSKPGVFSFLDPLAYEIWMCIV
ATGLR1.1   KYDAAVGDITITSNRSLYVDFTLPYTDIGIGILTVKKKSQ..GMWTFFDPFEKSLWLASG
ATGLR1.2   KYDAAVGDITITSDRSMYVDFTLPYTEMGLGIVAAKERS....MWVFFQPLTPNLWITSA
ATGLR1.3   KYDAAVGDITITSNRSTYVDFTLPFTEMGLGIVAVKERS....MWVFFQPLTPDLWITSA
ATGLR1.4   KYDAAVGDITITDNRSLYVDFTLPFTDMGLAVVTAKDKS....MWIIFKPLTLSLWLTIA
ATGLR2.1   KYDAVVADTTISSNRSMYVDFSLPYTPSGVGLVVPVKDSVRRSSTIFLMPLTLALWLISL
ATGLR2.2   QFDAVVGDTTILANRSSFVDFTLPFMKSGVGLIVPLKDEVKRDKFSFLKPLSIELWLTTL
ATGLR2.3   RYDAVVGDTTILVNRSSYVDFTFPFIKSGVGLIVEMTDPVKRDYILFMKPLSWKLWLTSF
ATGLR2.4   VFDAVVGDTTILANRSSYVDFTLPYTTSGVGMVVPLKDNVARSSLIFFKPLTPGLWGMTL
ATGLR2.5   EFDGAVGDTTILANRSHYVDFALPYSETGIVFLVPVKDGKEKGEWVFLKPLTKELWLVTA
ATGLR2.6   EFDGAVGDTTILANRSTYVDFALPYSETGIVVVVPVKDEREKGKWVFLKPLTRELWFLTA
ATGLR2.7   AYDAVVGDVTIVANRSLYVDFTLPYTESGVSMMVPLKDN..KNTWVFLRPWSLDLWVTTA
ATGLR2.8   TLDAVVGDVTITAYRSLYADFTLPYTESGVSMMVPVRDNENKNTWVFLKPWGLDLWVTTA
ATGLR2.9   TWDAVVGDITITANRSLYADFTLPFTESGVSMMVPVRDNENKDTWVFLEPWSLELWVTTG
ATGLR3.1   .FDAVVGDIAIVTKRTRIVDFTQPYIESGLVVVAPVTRLNE.NPWAFLRPFTLPMWAVTA
ATGLR3.2   .FDAVVGDIAIVTKRTRIVDFTQPYIESGLVVVAPVTKLND.TPWAFLRPFTPPMWAVTA
ATGLR3.3   .FDGVVGDVAIVTNRTKIVDFTQPYAASGLVVVAPFKKLNS.GAWAFLRPFNRLMWAVTG
ATGLR3.4   .FDVAVGDITIVTNRTRYVDFTQPFIESGLVVVAPVKEAKS.SPWSFLKPFTIEMWAVTG
ATGLR3.5   .FDVAVGDVTIITNRTKFVDFTQPFIESGLVVVAPVKGAKS.SPWSFLKPFTIEMWAVTG
ATGLR3.6   .YDAGVGDITIITERTKMADFTQPYVESGLVVVAPVRKLGS.SAMAFLRPFTPQMWLIAA
ATGLR3.7   .YDAAVGDIAIVPSRSKLVDFSQPYASTGLVVVIPANDDN..ATWIFLRPFTSRLWCVVL
```

```
              340                    350                  360        370
GhGLR4.8   AMTMFNAFVIWLV.............ESRTGHESVG........AIFWFPLATLFYGGHR
GluA2      FAYIGVSVVLFLVSRFSPYEWHTEEFEDGRETQSSESTNEFGIFNSLWFSLGAFMQQGCD
ATGLR1.1   AFFVLTGIVVWLV.............ERSVNPEFQGSWG.QQLSMMLWFGFSTIVFA.HR
ATGLR1.2   AFFVLTGIIVWLI.............ERAENKEFQGSWP.QQIGVVIWFGFSTLVYA.HR
ATGLR1.3   FFFVLTGVIVWLI.............ERAENKEFQGSWP.QQIGVVLWFGFSTLVYA.HR
ATGLR1.4   SFFILTGAIVWLI.............ERHDNADFQGSCF.QQIGTLLCFGFSTLVFA.HR
ATGLR2.1   LSFFIIGLVVWVL.............EHRVNPDFDGPGQ.YQLSTIFWFSFSIMVFA.PR
ATGLR2.2   VFFFLVGISVWTL.............EHRVNSDFRGPAN.YQASTIFWFAFSTMVFA.PR
ATGLR2.3   ISFFLVGCTVWVL.............EYKRNPDFSGPPR.FQASTICWFAFSTMVFA.PR
ATGLR2.4   GSFFVVGFVVWIL.............EHRVNSEFTGPPQ.YQISTMFWFAFSIMVFA.PR
ATGLR2.5   ASFLYIGIMVWIF.............EYQADEEFREQMIIDKISSVFYFSFSTLFFA.HR
ATGLR2.6   ASFLYIGIM...................................................
ATGLR2.7   CFFVFIGFIVWIL.............EHRVNTDFRGPPH.HQIGTSFWFAFSTMNFA.HR
ATGLR2.8   CFFVLIGFVVWLF.............EHRVNTDFRGPPH.HQIGTSFWFSFSTMVFA.HR
ATGLR2.9   CFFVFIGFVVWLF.............EHRVNTDFRGPPQ.YQIGTSLWFSFSTMVFA.HR
ATGLR3.1   SFFVIVGAAIWIL.............EHRINDEFRGPPR.RQIITILWFTFSTMFFS.HR
ATGLR3.2   AFFLIVGSVIWIL.............EHRINDEFRGPPR.KQIVTILWFSFSTMFFS.HR
ATGLR3.3   CCFLFVGIVVWIL.............EHRTNDEFRGPPK.RQCVTILWFSFSTMFFA.HR
ATGLR3.4   GFFLFVGAMVWIL.............EHRFNQEFRGPPR.RQLITIFWFSFSTMFFS.HR
ATGLR3.5   ALFLFVGAVIWIL.............EHRFNEEFRGPPR.RQIITVFWFSFSTMFFS.HR
ATGLR3.6   ASFLIVGAVIWCL.............EHKHNDEFRGPPR.RQVITTFWFSFSTLFFS.HR
ATGLR3.7   VSFLVIAVVIWIL.............EHRINEDFRGPPR.RQLSTMLLFSFSTLFKR.NQ
```

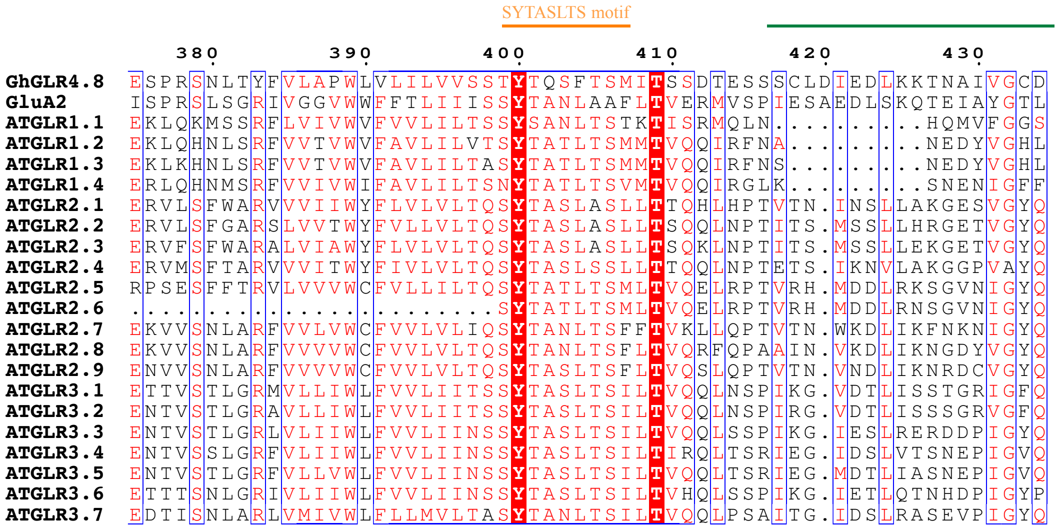
SYTASLTS motif
380 390 400 410 420 430
GhGLR4.8
GluA2
ATGLR1.1
ATGLR1.2
ATGLR1.3
ATGLR1.4
ATGLR2.1
ATGLR2.2
ATGLR2.3
ATGLR2.4
ATGLR2.5
ATGLR2.6
ATGLR2.7
ATGLR2.8
ATGLR2.9
ATGLR3.1
ATGLR3.2
ATGLR3.3
ATGLR3.4
ATGLR3.5
ATGLR3.6
ATGLR3.7

S2

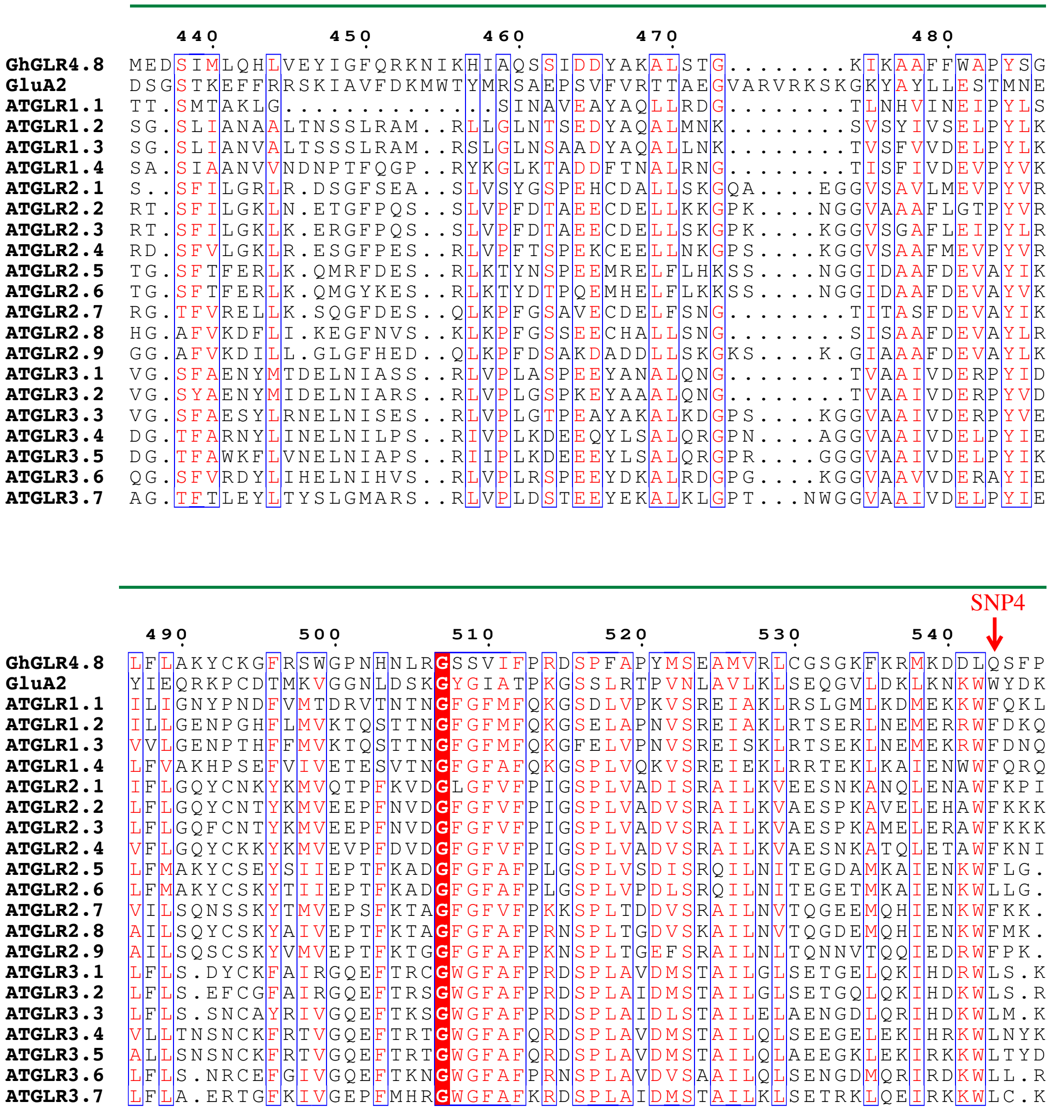
440 450 460 470 480
490 500 510 520 530 540
SNP4
GhGLR4.8
GluA2
ATGLR1.1
ATGLR1.2
ATGLR1.3
ATGLR1.4
ATGLR2.1
ATGLR2.2
ATGLR2.3
ATGLR2.4
ATGLR2.5
ATGLR2.6
ATGLR2.7
ATGLR2.8
ATGLR2.9
ATGLR3.1
ATGLR3.2
ATGLR3.3
ATGLR3.4
ATGLR3.5
ATGLR3.6
ATGLR3.7

```
           550                        560       570       580       590
GhGLR4.8   ECSSST...........IDVTMKR.....GIGPGPFSGLFILSGTASAVAILITVIRPMR
GluA2      GECGAKD........SGSKEKTS......ALSLSNVAGVFYILVGGLGLAMLVALIEFCY
ATGLR1.1   DSLNVHSNTEEVASTNDDDEASK......RFTFRELRGLFIIAGAAHVLVLALHLFHTRQ
ATGLR1.2   LP.............YTTDDTSN......PITLYRFRGLFMITGVSFAFALAVLLILWLR
ATGLR1.3   LP.............YTTDDTSN......PITLYRFRGLFIIIGVSFAFALAVLVILCLR
ATGLR1.4   TTS............ATSEDTFH......PLTVYTFRGLFMITGVSFAFALIVYLIPWNR
ATGLR2.1   DESCPD.........PLTNPDPNPSVSFRQLGFDSFWVLFLVAAIVCTMALLKFVYQFLK
ATGLR2.2   EQSCPD.........PVTNPDSNPTVTAIQLGVGSFWFLFLVVFVVCVLALGKFTFCFLW
ATGLR2.3   EQSCPD.........PITNPDPNPSFTSRQLDIDSFLFLFVGVLLVCVMALGNFTYCFLA
ATGLR2.4   DKTCPD.........PMNNPDPNPTVSFRKLSLDSFLLLFVAAATVCTLALLKFVICFLI
ATGLR2.5   EKHCLD.........STTSDSP......IQLDHHSFEALFLIVFVVSVILLLLMLASRGY
ATGLR2.6   EKHCLD.........STTSDSP......IRLDHHSFEALFTIVFVVSMLLLLAMLVCRRY
ATGLR2.7   PNNCPD.........LNTSLSSN......HLSLSSFWGLFLIAGIASFLALLIFVANFLY
ATGLR2.8   QNDCPD.........PKTALSSN......RLSLRSFWGLFLIAGIASFLALLIFVFLFLY
ATGLR2.9   KNDCPD.........PMTALSSN......RLNLSSFLGLFLIAGTAISFSLLVFVALFLY
ATGLR3.1   SNCSSPHG.......SQSG.DSE......QLNVHSFWGMFLVVGIACLVALFIHFFKIIR
ATGLR3.2   SNCSNLNG.......SVSDEDSE......QLKLRSFWGLFLVCGISCFIALFIYFFKIVR
ATGLR3.3   NACTLEN..........AELESD......RLHLKSFWGLFLICGVACLLALFLYFVQIIR
ATGLR3.4   HECSMQI..........SNSEDS......QLSLKSFWGLFLICGITCFMALTVFFWRVFW
ATGLR3.5   HECTMQI..........SDTENY......QISVQSFWGLFLICGVVWFIALTLFCWKVFW
ATGLR3.6   KACSLQG..........AEIEVD......RLELKSFWGLFVVCGVACVLALAVYTVLMIR
ATGLR3.7   TNCAGKSN.........WNPEPN......QLHLKSFKGLYLVCIAITVSAFLVFVLRMIR
```

```
                                  600
GhGLR4.8   RRWER................LVQGMLMG..........................RGLWV
GluA2      KSRAEAKR............MKVAKNAQN..........................INPSS
ATGLR1.1   EVSRLCTK..............LQSFYK................................
ATGLR1.2   ERWEILVNS.....VNIYFSQRLRHFRILFTR.......................TIHPS
ATGLR1.3   DKWEILVD.......NLDLSQRLRHFRIHFVR.......................SIHTS
ATGLR1.4   EQRQVVLK............HFHRYVSHRFAR.......................EIRPS
ATGLR2.1   EN...............PNQRNLRVLWEKFN.......................EPDQKS
ATGLR2.2   KT...................KGKDLWKEFL.......................KRDTDS
ATGLR2.3   K.......................................................DQVS
ATGLR2.4   QNRIILND.......EFYRGKRMKEMWLKFM.......................ESDGES
ATGLR2.5   QERQHN...............ASPNLPNDQ........................ANAAQE
ATGLR2.6   RQESKSGEINANNSPTDGNMRAPPNQPTDDNMRAPTSPPIDDQVLEPPGPALNEADDQDQ
ATGLR2.7   EHKHTLFD.....DSENSFRGKLKFLVRNFDEKDIKSHMFKENAVHNVSSPITQGSSSPL
ATGLR2.8   ENRHTLCD.....DSEDSIWRKLTSLFRNFDEKDIKSHTFKSSAVHHVSSPMTQYIPSPS
ATGLR2.9   EHRHTLGD.....DSEDSLWRKLKFLFKIFDEKDMNSHTFKNSAIHNISSPMTHKTPSPS
ATGLR3.1   DFCKDTP............EVVVEEAIPS...PK....................SSRLTK
ATGLR3.2   DFFRHG.............KYDEEATVPS...PE....................SSRSKS
ATGLR3.3   QLYKKPT............DDAIARDQQQNHDSS....................SMRSTR
ATGLR3.4   QYQRLLP............ESADEERAGEVSEPSRSG................RGSRAPS
ATGLR3.5   QYQRLRP............EESDEVQARSEEAGSSRG................KSLRAVS
ATGLR3.6   QFGQQCP............EEAEGSIRRR.........................SSPSAR
ATGLR3.7   QFVRYRR............MERTSSMPRASWSASP..................TLRLREL
```

```
          610       620       630       640
GhGLR4.8   WLTTLFSRDQRGNQLQVQLARISFTSQTLTSS....................
GluA2      SQNSQNFATYKEGYNVYGIESVKI............................
ATGLR1.1   ....................................................
ATGLR1.2   PLGLDNPIGENAVQMAQRNRR...............................
ATGLR1.3   PL..DDPIGETAVQMAQQNRQ...............................
ATGLR1.4   PT.TPNRQNENSVI......................................
ATGLR2.1   YIKDVTKCQCSSGQGMPKNGQEGAN............AVNNGN.........
ATGLR2.2   YINDIEKCLCSQEMPENSNKATNQTNYGMELRVRNIVQVNQTDPDCL.....
ATGLR2.3   YLDKVEMSPCSSSQQMPVKRKTQLN..........MSQVHDQDSL.......
ATGLR2.4   YIS...RVRSTCPQVLIQPREEDID............PING...........
ATGLR2.5   EVNEEGNVG.....DHIVEVDTALVRRK..KLTSNTIPIRRVAPLSRLKSA.
ATGLR2.6   LLNDEVNVGDRNEVDIIVEVDPTLVHRRN.LITSKTIPTRRAALFSRIKSA.
ATGLR2.7   TDQSTPLPRSPEQYRELELRRVSSISSGELFTTQ.SEQVEDEESAIIQCEGE
ATGLR2.8   TLQIAPRPHSPSQDRAFELRRVSFTPNEERLTTQ.TIHFEDEESDIECVVEQ
ATGLR2.9   TVQITPWPQSPSQNREFELRRVSFSPSEERFTTQPIIHHEDGESDIECRVEQ
ATGLR3.1   LQTFLAFVDEKEEETKRRLKRKRNNDHSMNANSIISRTASRRPI........
ATGLR3.2   LQTFLAYFDEKEDESKRRMKRKRNDDLSLKPS.........RPI........
ATGLR3.3   LQRFLSLMDEKEESKHESKKRKIDGSMNDTSGSTRSRGFDRERSFNSVNPLD
ATGLR3.4   FKELIKVVDKREAEIKEILKQKSSKKLKSTQSAAGTSQSQHGEIT.......
ATGLR3.5   FKDLIKVVDKREAEIKEMLKEKSSKKLKDGQSSAENSQSKDHETPQ......
ATGLR3.6   IHSFLSFVKEKEEDAKARSSRERQLEDISANGSSRCN...............
ATGLR3.7   VFDFVEFVDEKEEAIKRMFRRSDDSNNNPSHVGEVQADTEVPRN........
```

Supplementary Figure 7. Amino acid sequence alignment of GhGLR4.8 with AtGLRs and GluA2. SNP1-SNP4 are marked by red arrow, S1 segment of LBD is indicated by red line, S2 segment of LBD is indicated by green line, the conserved 'SYTASLTS' motif of M3 is indicated by orange line, and the number indicates the amino acid length of GhGLR4.8. The amino acid sequence from top to bottom is GhGLR4.8, GluA2 and *Arabidopsis* 20 AtGLRs.

Supplementary Figure 8

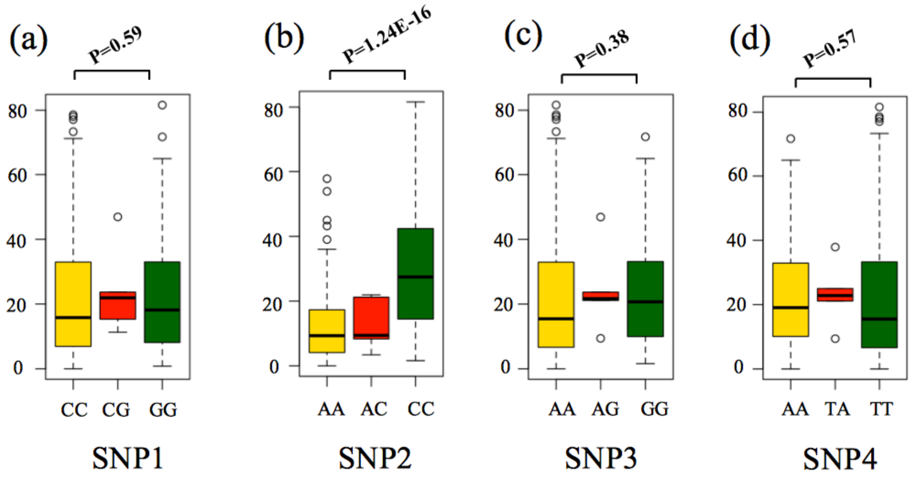

Supplementary Figure 8. Association analysis between FW resistance and four nucleotide variations in the *GhGLR4.8* coding sequence. **(a), (c) and (d)** Other three polymorphisms in *GhGLR4.8* are not associated with disease index. In box plots, center line indicates median, box limits denote upper and lower quartiles, and points indicate outliers. Statistical significance was determined by a two-sided *t*-test. **(b)** The disease index shows significant difference between allele 'AA' and allele 'CC' of the strongest association SNP (D03_2176763).

Supplementary Figure 9

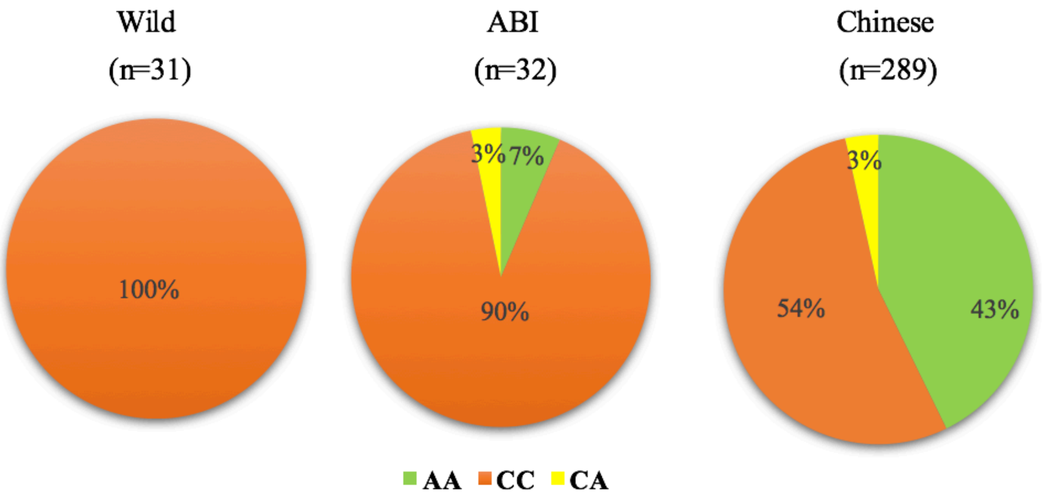

Supplementary Figure 9. Genotype frequency distribution of SNP D03_2176763. The SNP information of 352 resequenced accessions is derived from our previous study.[12] Wild group, wild cottons; ABI group, cottons from America, Brazil and India; Chinese group, cottons from China.

Supplementary Figure 10

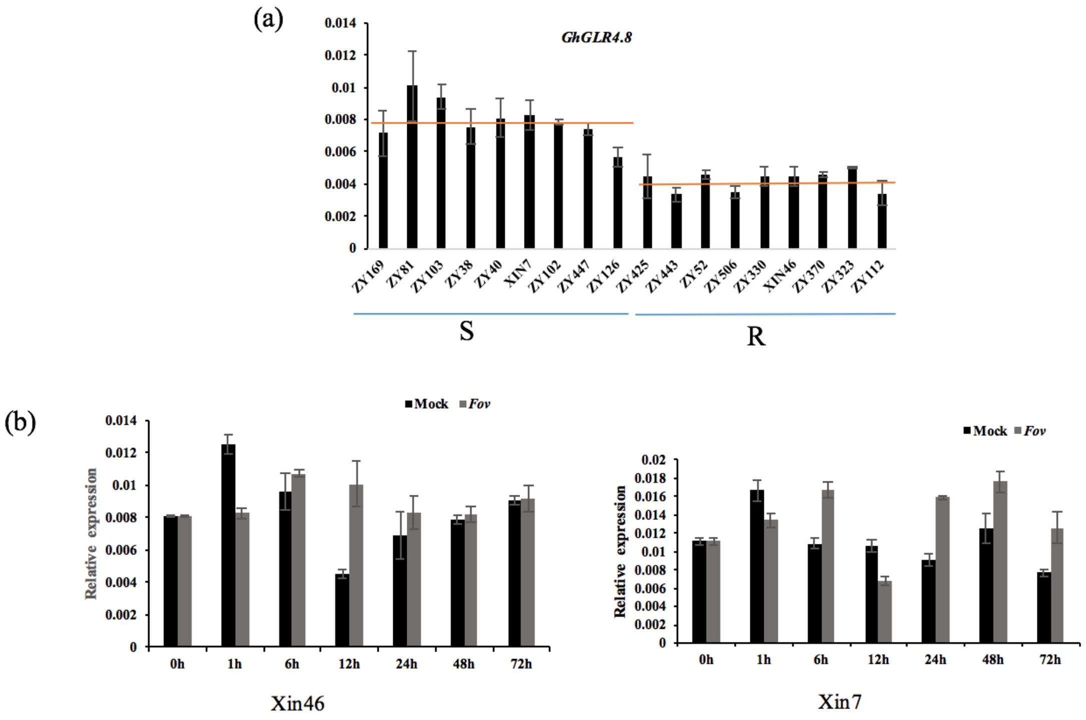

Supplementary Figure 10. The expression pattern of *GhGLR4.8* gene. **(a)** qRT-PCR analysis of the expression levels of *GhGLR4.8* in resistance (R) and susceptible (S) cotton varieties **(b)** qRT-PCR analysis of the expression levels of *GhGLR4.8* in resistant and susceptible cotton varieties at different time points after inoculation. The expression level of genes was normalized by the expression of *G. hirsutum UB7*, data are presented as mean ± SD from three technical replicates.

Supplementary Figure 11

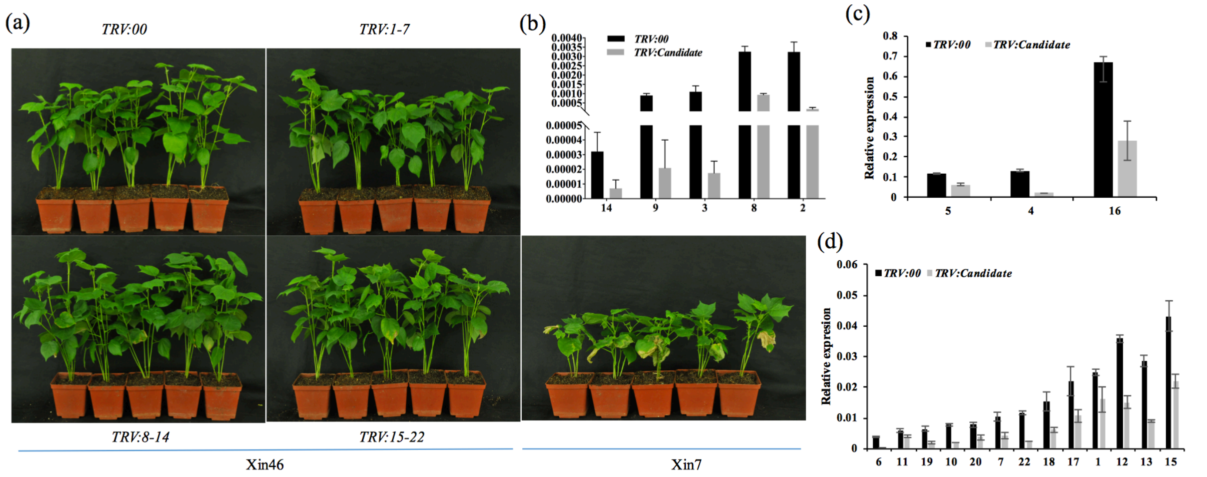

Supplementary Figure 11. Knockdown of candidate genes through VIGS except for *GhGLR4.8* has no effect on FW resistance. **(a)** Phenotype of *TRV:00* plants and genes-silenced plants at 20 days after inoculation with *Fov*. VIGS assay was conducted in a resistant cultivar Xinluzao 46 (Xin46) background. Susceptible cultivar Xinluzao 7 (Xin7) was inoculated with *Fov* simultaneously. **(b-d)** Detection of genes silencing efficiency through qRT-PCR. Data in b, c and d are presented as mean ± SD from three technical replicates.

Supplementary Figure 12

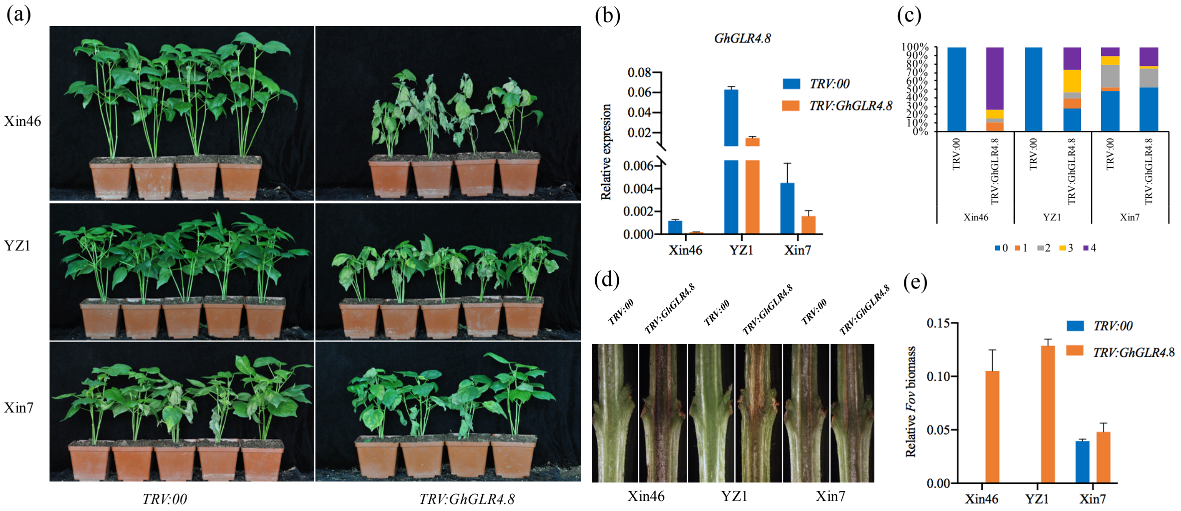

Supplementary Figure 12. Knockdown of *GhGLR4.8* strongly suppressed resistance to *Fov* in Upland cotton. **(a)** Disease symptoms of *TRV:00* and *TRV:GhGLR4.8* plants at 20 days after inoculation with *Fov*. Two highly resistant cultivars (Xin46 and YZ1) and one susceptible cultivars (Xin7) were selected. **(b)** Expression levels of *GhGLR4.8* in *TRV:00* and *TRV:GhGLR4.8* plants was determined by qRT-PCR. **(c)** Distribution of disease grade of *TRV:00* and *TRV:GhGRL4.8* plants. **(d)** Vascular bundles coloration in longitudinal-sections of inoculated *TRV:00* and *TRV:GhGLR4.8* plants. **(e)** Relative content of *Fov* DNA in inoculated stem of *TRV:00* and *TRV:GhGLR4.8* plants. Data in b and e are presented as mean ± SD from three technical replicates.

Supplementary Figure 13

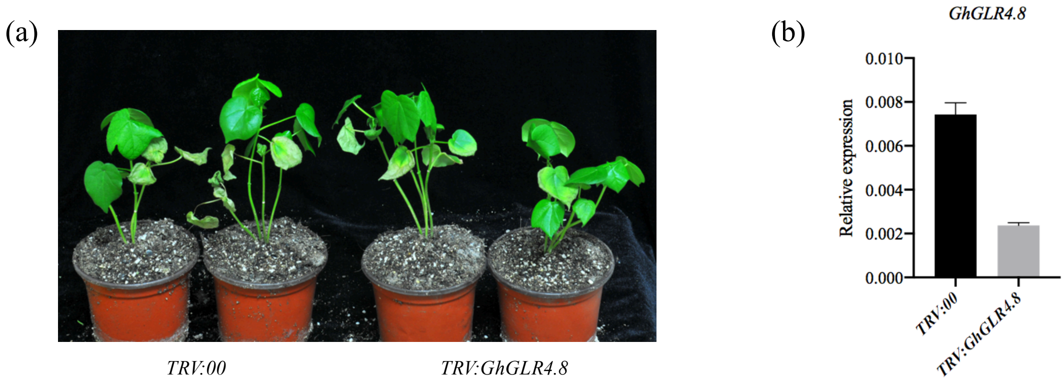

Supplementary Figure 13. Knockdown of *GhGLR4.8* through VIGS has no effect on Verticillium wilt resistance. **(a)** Disease symptoms of *TRV:00* and *TRV*:*GhGLR4.8* plants at 20 days after inoculation with *V. dahliae*. **(b)** Expression levels of *GhGLR4.8* in *TRV:00* and *TRV:GhGLR4.8* plants were determined by qRT-PCR. Data in b are presented as mean ± SD from three technical replicates.

Supplementary Figure 14

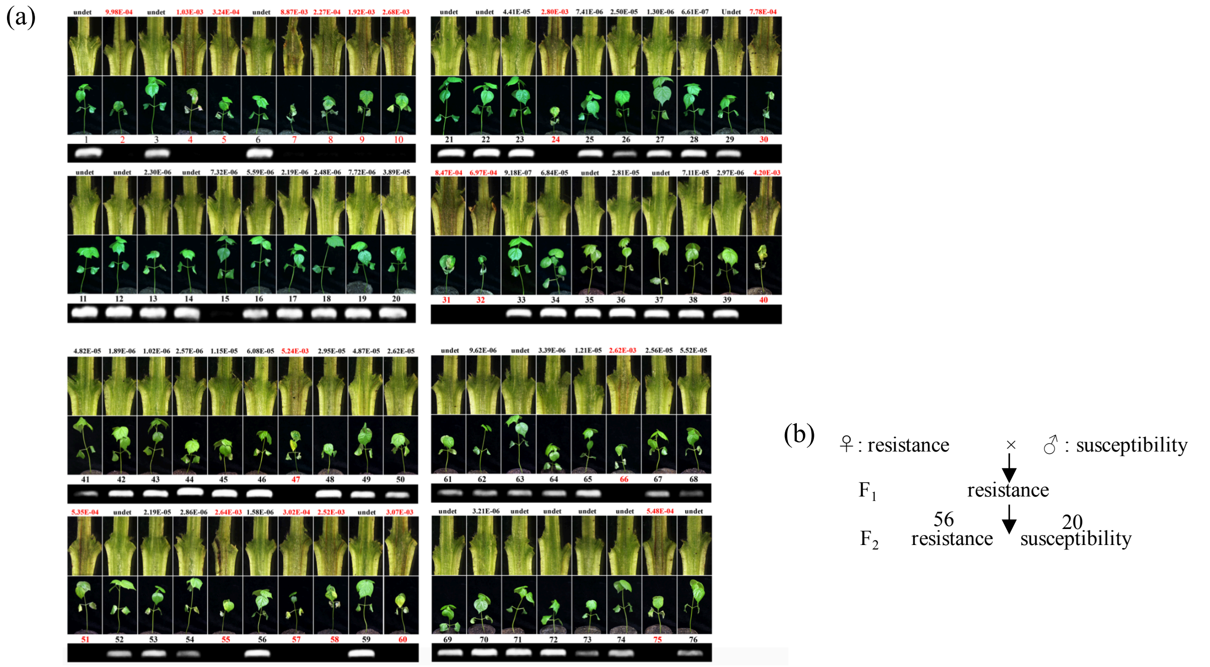

Supplementary Figure 14. Repeat of co-segregation analysis between *GhGLR4.8*$^{SNP(A/C)}$ genetic marker and *Fov* resistance. The detailed description refers to Figure 3.

Supplementary Figure 15

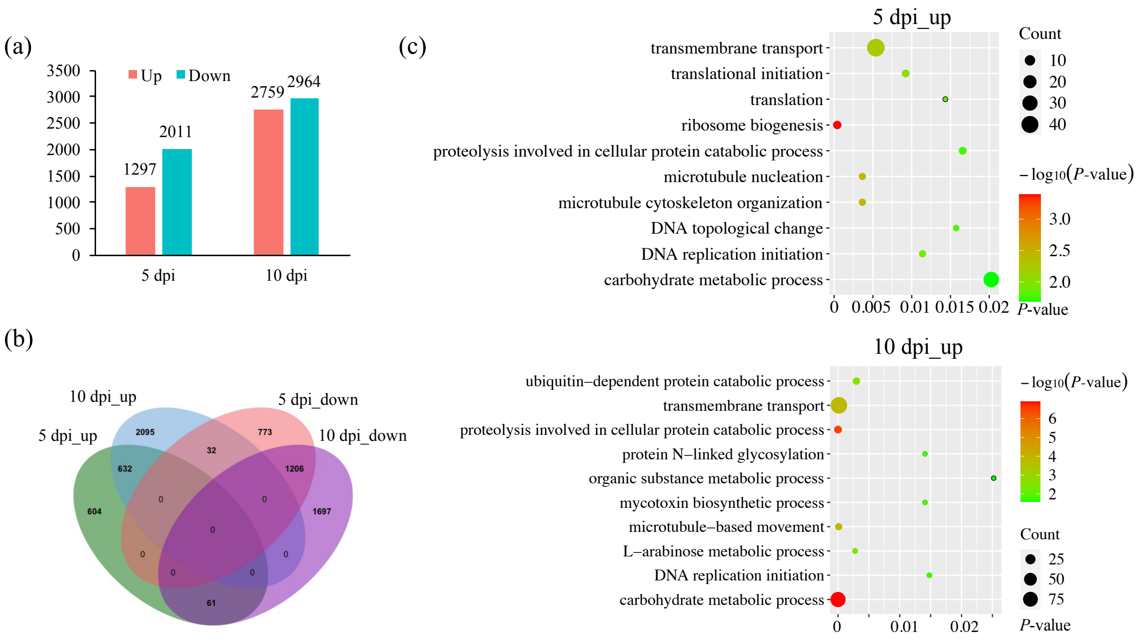

Supplementary Figure 15. Transcriptome profile of fungal genes in hypocotyls of *Fov*-infected *Fov7*_KO#5 versus *in vitro*-grown *Fov*. **(a)** The number of differentially expressed fungal genes (DEFGs) ($P < 0.05$, | log2 (FC) | > 1). Fold change was calculated by (*in planta Fov*)/(*in vitro*-grown *Fov*). **(b)** The venn diagram of DEFGs. **(c)** GO enrichment of host-induced genes of *Fov*. TOP10 significantly enriched biological process GO terms are show. Three biological replicates were included for each treatment.

Supplementary Figure 16

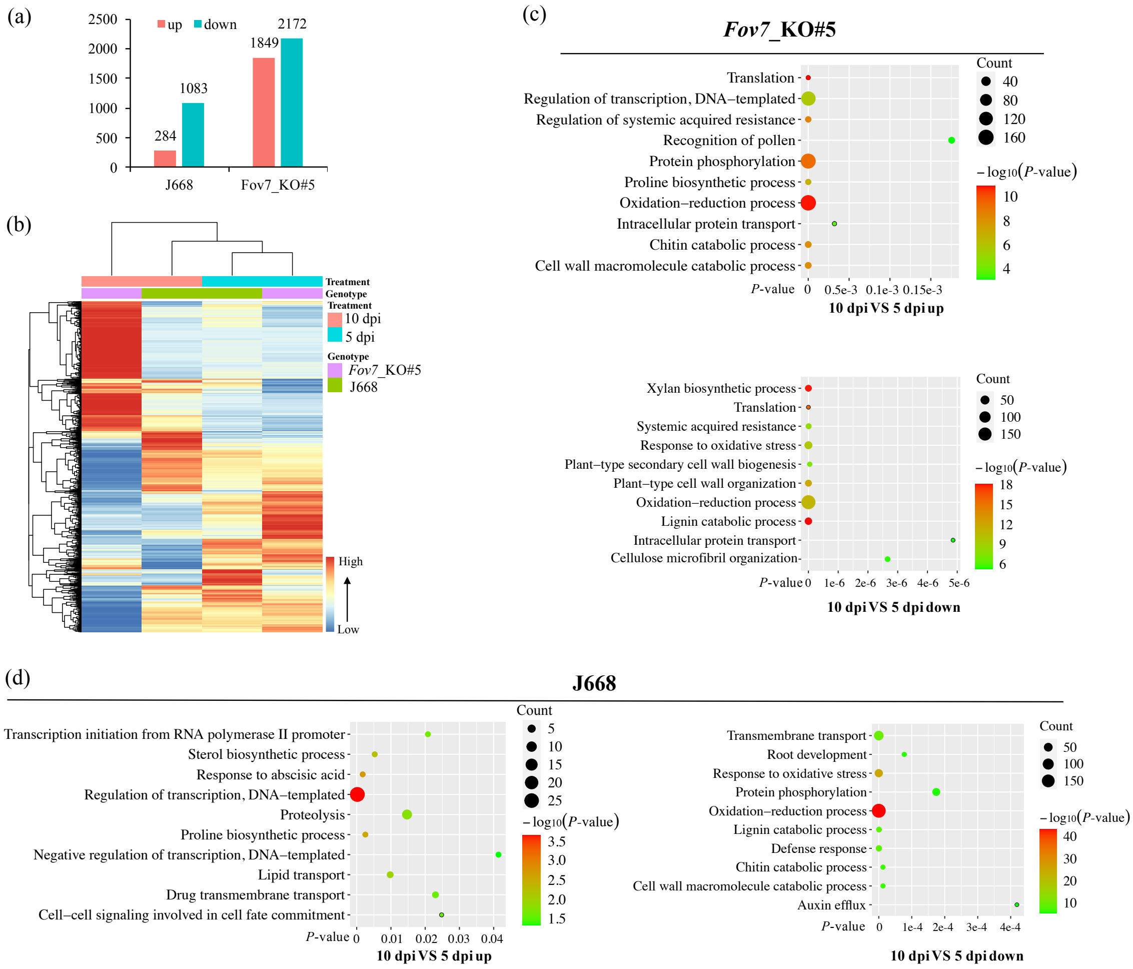

Supplementary Figure 16. Transcriptome profile of plant genes in hypocotyls of *Fov*-infected *Fov7*_KO#5 and J668 at 10 dpi versus at 5 dpi. **(a)** Number of differentially expressed plant genes (DEPGs) ($P < 0.05$, $|\log2(FC)| > 2$) in *Fov*-infected hypocotyls of *Fov7*_KO#5 and J668 at 10 dpi versus 5 dpi. Fold change is calculated by 10 dpi/5 dpi, dpi, days post inoculation. **(b)** Heat map of DEPGs in hypocotyls of *Fov7*_KO#5 and J668 at 10 dpi versus 5 dpi. **(c)** and **(d)** Gene ontology (GO) enrichment analysis of DEPGs in hypocotyls of *Fov7*_KO#5 **(c)** or J668 **(d)** at 10 dpi versus 5 dpi. Three biological replicates were included for each treatment.

Supplementary Figure 17

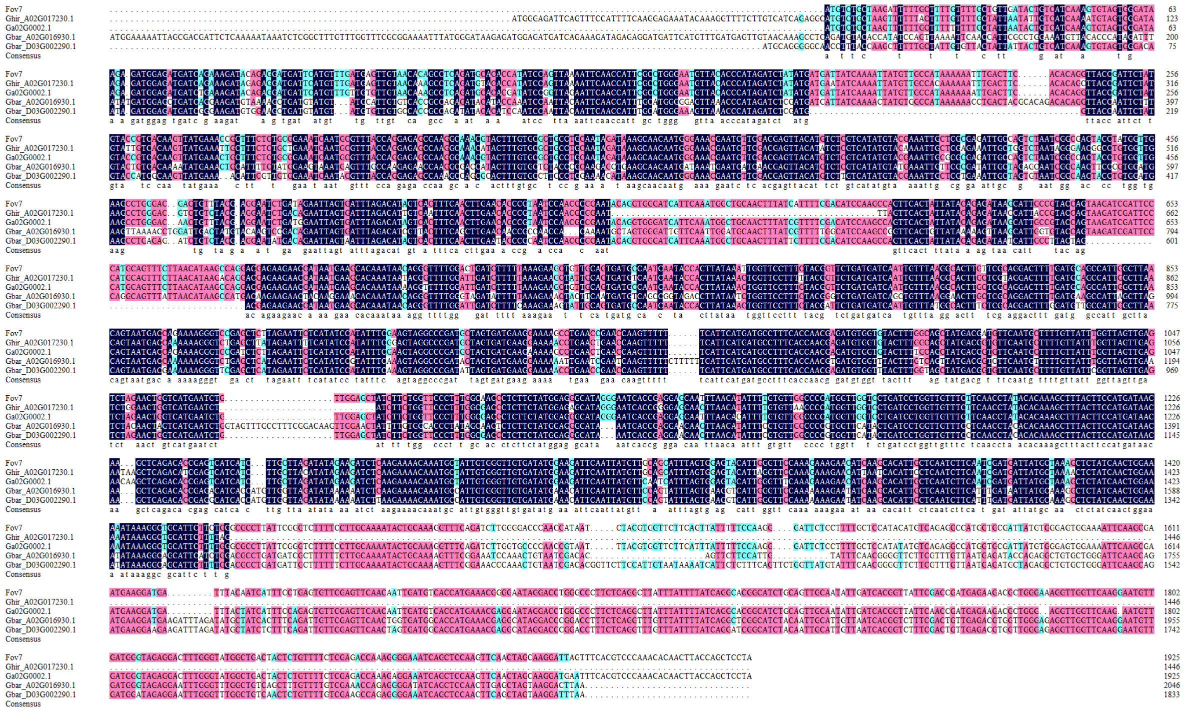

Supplementary Figure 17. Sequence comparison of *Fov7* in *G. hirsutum* (TM-1), [19] *G. barbadense* (3-79)[19] and *G. arboretum* (Shixiya1).[10] *Ghir_A02G017230.1* is the *Fov7* homologue in A subgenome of TM-1, *Ga02G0002.1* is the *Fov7* homologue in Shixiya1, *Gbar_A02G016930.1* and *Gbar_D03G002290.1* are the *Fov7* homologues in A and D subgenome of 3-79, respectively.

Supplementary Table

Supplementary Table 1, 3, 7-10 were offered in a separate file.

Supplementary Table 1. The disease index of 290 cotton accessions for GWAS.

Supplementary Table 2. The significant SNPs associated with Fusarium wilt.

SNP	Chromosome	Allele	Position	*P* value	-log10 (*P*)	MarkerR^2
D03_2176763	D03	G/T	2176763	7.84E-11	10.106	0.17535
A01_30018512	A01	A/T	30018512	6.47E-09	8.189	0.12425
D03_2135880	D03	C/T	2135880	9.11E-09	8.040	0.13716
D03_2146908	D03	G/A	2146908	1.81E-08	7.742	0.13175
D03_2115717	D03	G/A	2115717	2.34E-08	7.631	0.12975
D03_2148731	D03	G/A	2148731	6.76E-08	7.170	0.12145
D03_2135218	D03	A/T	2135218	7.34E-08	7.134	0.1208
D03_2115674	D03	C/T	2115674	9.72E-08	7.012	0.11862
D13_41766280	D13	T/G	41766280	1.35E-07	6.869	0.11606
D03_2125319	D03	A/G	2125319	1.54E-07	6.813	0.11506
D03_2076487	D03	A/C	2076487	1.78E-07	6.749	0.11393
D03_2380413	D03	T/G	2380413	2.04E-07	6.691	0.11288
D03_2145709	D03	G/A	2145709	2.32E-07	6.634	0.11187
D03_2147303	D03	C/T	2147303	2.79E-07	6.554	0.11045
D03_2138339	D03	A/G	2138339	2.99E-07	6.524	0.10991
D03_2073788	D03	G/A	2073788	3.40E-07	6.468	0.10893
D03_2075791	D03	G/A	2075791	3.52E-07	6.453	0.10866

Supplementary Table 3. The FW resistance of 194 cotton accessions collected from previous study.

Supplementary Table 4. Genes located in candidate region of *Fov7* locus and its annotation.

Gene ID	Strand	Start	Stop	Annotation
Gh_D03G0203	-	2027606	2029122	heat shock factor 4
Gh_D03G0204	+	2045424	2045591	unknown
Gh_D03G0205	+	2094618	2094950	chloride channel B
Gh_D03G0206	-	2125102	2144777	cytochrome P450, family 83
Gh_D03G0207	-	2166718	2167364	Protein of unknown function (DUF1685)
Gh_D03G0208	-	2169915	2173932	unknown
Gh_D03G0209	-	2175025	2177322	glutamate receptor 3.3
Gh_D03G0210	+	2179082	2182850	unknown
Gh_D03G0211	+	2193477	2194673	unknown
Gh_D03G0212	-	2203285	2203968	bHLH DNA-binding superfamily protein
Gh_D03G0213	+	2218541	2219557	mitogen-activated protein kinase kinase kinase 19
Gh_D03G0214	+	2226884	2228258	F-box/RNI-like superfamily protein
Gh_D03G0215	-	2229735	2231887	PLC-like phosphodiesterases superfamily protein
Gh_D03G0216	-	2232858	2235938	Plant protein of unknown function (DUF869)
Gh_D03G0217	-	2238291	2240938	protein serine/threonine kinases
Gh_D03G0218	-	2255453	2257785	Integrase-type DNA-binding superfamily protein
Gh_D03G0219	+	2293920	2297367	Cystathionine beta-synthase (CBS) family protein
Gh_D03G0220	-	2304123	2304617	DREB and EAR motif protein 3
Gh_D03G0221	-	2314748	2317694	Dentin sialophosphoprotein-related
Gh_D03G0222	+	2338597	2347155	P-loop containing nucleoside triphosphate hydrolases
Gh_D03G0223	-	2348736	2351761	Leucine-rich receptor-like protein kinase family protein
Gh_D03G0224	+	2368534	2373145	2-oxoglutarate (2OG) and Fe(II)-dependent oxygenase
Gh_D03G0225	-	2373295	2374910	FMN-linked oxidoreductases superfamily protein

Supplementary Table 5. Features of the *GLR* genes in Upland cotton

Gene ID	Gene Name	Chr	Length (aa)	Genomic Region
Ghir_A07G021120	GhGLR1.1	A07	627	91819388-91821699
Ghir_A07G021130	GhGLR1.2	A07	847	91850132-91854091
Ghir_D07G009840	GhGLR1.3	D07	792	11852666-11858516
Ghir_D07G021310	GhGLR1.4	D07	862	53661343-53664759
Ghir_D07G021320	GhGLR1.5	D07	776	53666922-53669913
Ghir_A07G003890	GhGLR2.1	A07	979	4330442-4336180
Ghir_D04G008970	GhGLR2.2	D04	851	18628789-18638112
Ghir_D04G015310	GhGLR2.3	D04	914	49556864-49564141
Ghir_D07G003910	GhGLR2.4	D07	915	4037638-4043561
Ghir_A02G019470	GhGLR3.1	A02	947	107934254-107939151
Ghir_A05G018230	GhGLR3.2	A05	851	17246944-17253044
Ghir_A12G001330	GhGLR3.3	A12	885	1683238-1688933
Ghir_A12G016770	GhGLR3.4	A12	813	91878901-91883964
Ghir_A13G006510	GhGLR3.5	A13	905	14068841-14073278
Ghir_A13G009800	GhGLR3.6	A13	821	52745568-52750576
Ghir_A13G009810	GhGLR3.7	A13	921	52799328-52804364
Ghir_D03G000100	GhGLR3.8	D03	310	98496-99510
Ghir_D05G018240	GhGLR3.9	D05	909	15825979-15832082
Ghir_D12G001360	GhGLR3.10	D12	810	1630516-1636164
Ghir_D12G017030	GhGLR3.11	D12	813	49647233-49652268
Ghir_D13G005830	GhGLR3.12	D13	810	7699695-7704665
Ghir_D13G009280	GhGLR3.13	D13	821	19596508-19601650
Ghir_D13G009290	GhGLR3.14	D13	921	19625438-19630396
Ghir_A02G017230	GhGLR4.1	A02	481	105680541-105683016
Ghir_A02G017250	GhGLR4.2	A02	566	105697917-105714265

Ghir_A05G016210	GhGLR4.3	A05	900	15273260-15279087
Ghir_A05G020820	GhGLR4.4	A05	405	20134953-20137186
Ghir_A10G002660	GhGLR4.5	A10	904	2350198-2354896
Ghir_A10G002670	GhGLR4.6	A10	972	2354914-2358484
Ghir_A10G002680	GhGLR4.7	A10	889	2361358-2365538
Ghir_D03G002390	GhGLR4.8	D03	641	2246112-2248669
Ghir_D05G016050	GhGLR4.9	D05	818	13966076-13971543
Ghir_D05G020760	GhGLR4.10	D05	740	18081772-18085238
Ghir_D10G003440	GhGLR4.11	D10	904	2872905-2876333
Ghir_D10G003450	GhGLR4.12	D10	932	2877535-2881153
Ghir_D10G003460	GhGLR4.13	D10	897	2882280-2888831

Supplementary Table 6. Percentage and number of unique RNA-seq reads aligned to *Fov* race 4.

			Days Post Inoculation			
			5	10	5	10
	Biological Replicate	Plant	Percentage of *Fov* Reads		Number of Uniquely Aligned Reads	
	1	*Fov7*_KO#5	0.10%	0.70%	17429	315378
	2	*Fov7*_KO#5	0.09%	0.69%	31558	201704
	3	*Fov7*_KO#5	0.06%	0.66%	13410	193339
Average			0.08%	0.68%	20799	236807

Supplementary Table 7. Transcriptome profiles of fungal genes in hypocotyls of *Fov*-infected *Fov7*_KO#5 versus *in vitro*-grown *Fov*, related to Figure S15.

Supplementary table 8. Transcriptome profiles of plant genes in hypocotyls of *Fov*-infected *Fov7*_KO#5 versus J668 at 5 dpi and 10 dpi, related to Figure 5.

Supplementary table 9. Transcriptome profiles of plant genes in hypocotyls of *Fov*-infected *Fov7*_KO#5 and J668 at 10 dpi versus 5 dpi, related to Figure S16.

Supplementary Table 10. Primers used in this study.
